# Supplementary material for: Soil fertility management for yield and income stability in soybean-wheat rotation system under climate variability in subtropical Central India
Source: PLoS One. 2026 Jan 21;21(1):e0341035. doi: 10.1371/journal.pone.0341035 (PMC12822991; doi:10.1371/journal.pone.0341035)
Supplement: Attachment — Submitted filename: PONE-D-25-53731_R1.pdf [file pone.0341035.s004.pdf]

PLOS ONE

Soil fertility management for yield and income stability in soybean–wheat rotation system under climate variability in subtropical Central India

--Manuscript Draft--

Manuscript Number:	PONE-D-25-53731R1
Article Type:	Research Article
Full Title:	Soil fertility management for yield and income stability in soybean–wheat rotation system under climate variability in subtropical Central India
Short Title:	Managing Soil Fertility for Soybean–Wheat Productivity
Corresponding Author:	SANJAY SRIVASTAVA<br>ICAR Indian Institute of Soil Science<br>Bhopal, Madhya Pradesh INDIA
Keywords:	Soil health, soybean-wheat system, site specific nutrient use, residue management
Abstract:	The soybean–wheat rotation system forms a crucial part of Central India's rainfed agriculture. While traditionally known for its productivity and profitability, the system's sustainability is increasingly threatened by suboptimal nutrient management, and soil degradation under changing climate. To examine how nutrient management practices and farmers' efficiency in utilizing on-farm resources influence yield and income in soybean–wheat rotation system under variable environmental conditions in India a three-year study was conducted in Central India using a Participatory Technology Development (PTD) approach. Four nutrient management strategies were tested on small to semi-medium farms: (T1) integrated nutrient management using chemical fertilizers, farmyard manure and biofertilizers, (T2) integrated nutrient management using chemical fertilizers, enriched compost and biofertilizers, (T3) soil test crop response-based fertilization, and (T4) farmers' conventional practice. Among the four treatments T2 achieved the highest system productivity (6.63 t ha^{-1} wheat equivalent), net returns (67,680 INR ha^{-1}), and yield stability, under climatic variability. T3 offered moderate productivity with balanced nutrient use and lower input costs. Both T2 and T3 enhanced microbial activity and soil health, while T4 led to nutrient depletion, especially potassium, and low returns. It is noted that the timing and distribution of monsoon rainfall significantly impacted performance and yield stability of soybean. This study highlights the critical need for integrated, site-specific, and farmer-centric nutrient management strategies to ensure sustainable productivity, profitability, and soil health under changing climatic conditions.
Order of Authors:	SHINOGI K.C.
	SANJAY SRIVASTAVA
	RADHA T.K.
	D.L.N. RAO
	BHARAT PRAKASH MEENA
	NISHANT KUMAR SINHA
	HIRANMOY DAS
	RASHMI I.
	ROSIN K.G.
	AMAR BAHADUR SINGH
	SANJIB KUMAR BEHERA
	MONORANJAN MOHANTY
Response to Reviewers:	A detailed, point-by-point response to reviewer comments has been uploaded as a separate file titled "Response to Reviewers".

Powered by Editorial Manager® and ProduXion Manager® from Aries Systems Corporation

Additional Information:	
**Question**	**Response**
**Financial Disclosure**<br><br>Enter a financial disclosure statement that describes the sources of funding for the work included in this submission. Review the submission guidelines for detailed requirements. View published research articles from *PLOS ONE* for specific examples.<br><br>This statement is required for submission and **will appear in the published article** if the submission is accepted. Please make sure it is accurate.<br><br>**Funded studies**<br>Enter a statement with the following details:<br>• Initials of the authors who received each award<br>• Grant numbers awarded to each author<br>• The full name of each funder<br>• URL of each funder website<br>• Did the sponsors or funders play any role in the study design, data collection and analysis, decision to publish, or preparation of the manuscript?<br><br>Did you receive funding for this work?	The author(s) received no specific funding for this work.
**Competing Interests**<br><br>Use the instructions below to enter a competing interest statement for this submission. On behalf of all authors, disclose any competing interests that could be perceived to bias this work—acknowledging all financial support and any other relevant financial or non-financial competing interests.<br><br>This statement is required for submission and **will appear in the published article** if the submission is accepted. Please make sure it is accurate and that any funding sources listed in your Funding Information	The authors have declared that no competing interests exist.

later in the submission form are also declared in your Financial Disclosure statement.<br><br>View published research articles from *PLOS ONE* for specific examples.<br><br>**NO authors have competing interests**<br><br>Enter: *The authors have declared that no competing interests exist.*<br><br>**Authors with competing interests**<br><br>Enter competing interest details beginning with this statement:<br><br>*I have read the journal's policy and the authors of this manuscript have the following competing interests: [insert competing interests here]*<br><br>* typeset	
**Ethics Statement**<br><br>Enter an ethics statement for this submission. This statement is required if the study involved:<br><br>• Human participants<br>• Human specimens or tissue<br>• Vertebrate animals or cephalopods<br>• Vertebrate embryos or tissues<br>• Field research<br><br>Write "N/A" if the submission does not require an ethics statement.<br><br>General guidance is provided below. Consult the submission guidelines for detailed instructions. **Make sure that all information entered here is included in the Methods section of the manuscript.**	N/A

**Format for specific study types**<br><br>**Human Subject Research (involving human participants and/or tissue)**<br>• Give the name of the institutional review board or ethics committee that approved the study<br>• Include the approval number and/or a statement indicating approval of this research<br>• Indicate the form of consent obtained (written/oral) or the reason that consent was not obtained (e.g. the data were analyzed anonymously)<br><br>**Animal Research (involving vertebrate animals, embryos or tissues)**<br>• Provide the name of the Institutional Animal Care and Use Committee (IACUC) or other relevant ethics board that reviewed the study protocol, and indicate whether they approved this research or granted a formal waiver of ethical approval<br>• Include an approval number if one was obtained<br>• If the study involved *non-human primates*, add *additional details* about animal welfare and steps taken to ameliorate suffering<br>• If anesthesia, euthanasia, or any kind of animal sacrifice is part of the study, include briefly which substances and/or methods were applied<br><br>**Field Research**<br><br>Include the following details if this study involves the collection of plant, animal, or other materials from a natural setting:<br>• Field permit number<br>• Name of the institution or relevant body that granted permission	
**Data Availability**<br><br>Authors are required to make all data underlying the findings described fully available, without restriction, and from the time of publication. PLOS allows rare exceptions to address legal and ethical concerns. See the PLOS Data Policy and FAQ for detailed information.	Yes - all data are fully available without restriction

A Data Availability Statement describing where the data can be found is required at submission. Your answers to this question constitute the Data Availability Statement and **will be published in the article**, if accepted.<br><br>**Important:** Stating 'data available on request from the author' is not sufficient. If your data are only available upon request, select 'No' for the first question and explain your exceptional situation in the text box.<br><br>Do the authors confirm that all data underlying the findings described in their manuscript are fully available without restriction?	
**Describe where the data may be found in full sentences. If you are copying our sample text, replace any instances of XXX with the appropriate details.**<br><br>• If the data are **held or will be held in a public repository**, include URLs, accession numbers or DOIs. If this information will only be available after acceptance, indicate this by ticking the box below. For example: *All XXX files are available from the XXX database (accession number(s) XXX, XXX.*).<br>• If the data are all contained **within the manuscript and/or Supporting Information files**, enter the following: *All relevant data are within the manuscript and its Supporting Information files.*<br>• If neither of these applies but you are able to provide **details of access elsewhere**, with or without limitations, please do so. For example:<br><br>*Data cannot be shared publicly because of [XXX]. Data are available from the XXX Institutional Data Access / Ethics Committee (contact via XXX) for researchers who meet the criteria for access to confidential data.*<br><br>*The data underlying the results presented in the study are available from (include the name of the third party*	All datasets used in producing the results are provided in the Supporting Information file.

Powered by Editorial Manager® and ProduXion Manager® from Aries Systems Corporation

and contact information or URL).<br>• This text is appropriate if the data are owned by a third party and authors do not have permission to share the data.<br><br>* typeset	
Additional data availability information:	

Manuscript Click here to access/download;Manuscript;Manuscript.docx

TITLE, AUTHOR, AFFILIATION

Soil fertility management for yield and income stability in soybean-wheat rotation system under climate variability in subtropical Central India

Shinogi K C[1], Sanjay Srivastava[1*], Radha T K[2], D L N Rao[1#], Bharat Prakash Meena[1], Nishant Kumar Sinha[1], Hiranmoy Das[3], Rashmi I[4], Rosin K G[5], A B Singh[1#], Sanjib Kumar Behera[1], Monoranjan Mohanty[1]

Author Names and Affiliations:

[1] ICAR-Indian Institute of Soil Science, Bhopal, Madhya Pradesh, India

[2] ICAR-Indian Institute of Horticultural Research, Bangaluru, Karnataka, India

[3] ICAR-Indian Institute of Vegetable Research, Varanasi, Uttar Pradesh

[4] ICAR-Indian Institute of Soil and Water Conservation RC Kota, Rajasthan, India

[5] ICAR-Indian Agricultural research Institute, New Delhi, India

[#] Retired

* Corresponding Author:

E-mail: sanjaysrivastava238@gmail.com

Abstract

The soybean–wheat rotation system forms a crucial component of Central India's rainfed agriculture. Although historically productive and profitable, the system's sustainability is increasingly threatened by suboptimal nutrient management, and soil degradation under changing climate. This three-year study, conducted on small and semi-medium farms in Central India using a Participatory Technology Development (PTD) approach, aimed to evaluate how diverse nutrient management strategies influence yield, profitability, and resource use efficiency. Four nutrient management strategies were tested on small to semi-medium farms: (T_1) Integrated Nutrient Management (INM) using chemical fertilizers, farmyard manure and biofertilizers, (T_2) Integrated Nutrient Management using chemical fertilizers, enriched compost and biofertilizers, (T_3) Soil test crop response-based fertilization, and (T_4) Farmers' conventional practice. Among the four treatments T_2 achieved the highest system productivity (6.63 t ha^{-1} wheat equivalent), net returns (67,680 INR ha^{-1}), and superior yield stability, under climatic variability. T_3 offered moderate productivity while promoting balanced nutrient use and lower input costs. Both T_2 and T_3 enhanced microbial activity and soil health, while T_4 led to nutrient depletion, (especially potassium), and low returns. Furthermore, the timing and distribution of monsoon rainfall significantly impacted performance and yield stability of soybean. This study highlights the critical need for integrated, site-specific, and farmer-centric nutrient management strategies to ensure sustainable productivity, profitability, and soil health under changing climatic conditions.

Introduction

Crop diversification through legume–cereal rotation is an effective soil fertility management technique practiced globally. As a low-cost and sustainable strategy, this rotation supports both ecological intensification and economic resilience in diverse agroecosystems, particularly for

smallholder and rainfed agriculture systems (Yigezu *et al*., 2019; Meena *et al*., 2019). Legumes contribute nitrogen (N)-rich residues with a low carbon-to-nitrogen (C: N) ratio, which significantly enhances the yield of subsequent cereal crops (Watson *et al.*, 2017; Morrison *et al*., 2018). Additionally, alternating legumes with cereals is regarded as a natural method of biofortification, improving the nutritional value of cereal grains (Zuo and Zhang, 2009).

Globally, legume–cereal rotation systems exhibit wide global diversity, reflecting variations in land suitability and the climatic adaptability of different legumes and cereals. According to Leff *et al*. (2004), wheat (*Triticum aestivum*) is the most widely cultivated cereal, while soybean (*Glycine max*) is the leading leguminous oilseed crop globally. The soybean–wheat rotation system is especially predominant in temperate and subtropical regions (Caviglia *et al*., 2011), owing to its high productivity, profitability, and compatibility with regional climate conditions.

In India, the soybean–wheat rotation system has been practiced on the black soils (Vertisols) of Central India since the early 1980s, following the introduction of soybean as a rainfed crop in Madhya Pradesh. This rotation system was initially highly profitable, contributing significantly to regional agricultural incomes (Srivastava *et al*., 2001; Behera *et al*., 2007; Reddy *et al*., 2015). However, in recent years, climate change has severely disrupted its stability. Increasingly erratic rainfall patterns, drought stress during critical reproductive stages, and the emergence of new pests and diseases have severely impacted soybean yields. The national average soybean yield has declined to around 1.0 t ha^{-1}, which is significantly below the global average of 2.76 t ha^{-1} (GoI, 2023; USDA, 2024).

Concurrently with climate change, adoption of higher seed rates than recommended and unscientific nutrient management practices have further reduced the profitability of soybean-wheat rotation system in India, despite wheat maintaining higher yields (Sharma, 2016). Furthermore, in-situ burning of mechanically harvested wheat residues before summer

ploughing has linked the soybean-wheat rotation system to environmental degradation. This practice deteriorates the soil by depleting organic matter and nutrients present in crop residues and destroying beneficial soil microorganisms (Jain *et al*., 2014; Reddy *et al*., 2014; Kumar e*t al*., 2015).

To enhance the sustainability of the soybean-wheat rotation system in India, a range of agricultural technologies have been integrated over the years, particularly to build system resilience by improving soil health to withstand biotic and abiotic stresses, and by promoting stress-tolerant crop varieties. In line with the findings of long-term fertilizer experiments by Kundu *et al.* (2007) in the soybean-wheat system, soil researchers have developed precise nutrient management practices. These include integrating organic and inorganic fertilizers, retention of crop stubble in the field either as soil cover or through soil incorporation, and the recycling of stubble into nutrient-rich composts (Reddy *et al.,* 2014; Reddy *et al.,* 2015). Additionally, to facilitate minimal soil disturbance during soybean sowing while retaining wheat stubble, customized machinery has been introduced to farmers (Kushwah *et al*., 2016).

Despite substantial efforts by researchers and government agencies, studies have shown that field-level adoption of many of the recommended practices for soybean-based systems remains insufficient to achieve their intended ecological and economic benefits (Dupare *et al*., 2019; Dubey *et al*., 2022).

Consequently, social researchers note that farmers' adoption decisions are not solely driven by economic profitability. Instead, they are significantly influenced by factors such as the time, labour, and effort required to implement the practices, as well as the perceived practicality and utility of the technologies within the specific context of individual farming systems. For example, in the case of crop residue incorporation, a farmer's environmental awareness and willingness to incorporate straw into their fields are key factors influencing adoption decisions (Sattler *et al*., 2010; Lu *et al*., 2020).

A pilot study conducted by the ICAR-Indian Institute of Soil Science to examine the diffusion and adoption of recommended nutrient management practices in the soybean-wheat rotations revealed wide variability in the on-farm resource base of smallholder farmers. It also underscored the critical role of farmer participation in the development and evaluation of agricultural technologies. Recognizing these complexities, a study was undertaken using a Participatory Technology Development (PTD) approach in the soybean–wheat rotation system of Madhya Pradesh for a period of three years. This research aimed to determine how nutrient management practices and the efficiency with which farmers utilize available on-farm resources significantly influence yield and income in the soybean–wheat rotation system under varying environmental conditions in India.

Materials and Methods

Study area, experimental design and treatments

The study was conducted in the Berasia region, situated in Bhopal district, Madhya Pradesh, India. Geographically, the region is located at 23.6830253°N and 77.4773669°E, with an elevation of 470 meters at the eastern edge of the Malwa Plateau in central India. The climate is classified as subtropical, with most of the rainfall occurring during the southwest monsoon season. The mean annual temperature is 25°C, and annual rainfall ranges between 850 mm and 1800 mm. Predominant cropping systems in the area include soybean–wheat and soybean–chickpea rotations, with maize and rice cultivated as rainfed crops in certain pockets. Most farmers belong to marginal to medium landholding categories (Government of India, 2020).

An initial survey revealed low economic profitability attributed to suboptimal nutrient management practices. Farmers typically applied only nitrogenous and phosphatic fertilizers, with farmyard manure (FYM) applications limited to 1.5-2 t ha^{-1} every 2-3 years based on availability. Although high yielding varieties such as soybean JS-355 (yield potential ~1.89 t

ha^{-1}) and wheat Lok 1 and HI8498 (*Malav Shakti*) (yield potential >4.5 t ha^{-1}) were adopted by the farmers crop yields were only 1.2 t ha^{-1} for soybean and 2.5 t ha^{-1} for wheat, which clearly indicated inadequate nutrient management. To identify optimal nutrient management for maximizing yield and economic returns, Participatory Technology Development (PTD) approach was implemented across nine selected farms. Selection criteria for farmers included managerial capability and willingness to allocate resources and support trials. Farms were also selected in such a way that they equally represented landholding categories small (1-2 ha), medium (2-4 ha), and semi-medium (4-10 ha) categories.

For the field trials, four nutrient management practices were defined for the soybean-wheat crop rotation system as follows.

1. T_1 (INM_{FYM}): Integrated Nutrient Management (INM) with recommended 5 t ha^{-1} FYM, *Rhizobium* biofertilizers (R33 & R34, 4 kg ha^{-1}), and 50% NPK for soybean; PGPR biofertilizers (P3 & P10, 4 kg ha^{-1}) and 75% NPK for wheat.
2. T_2 (INM_{EC}): INM developed in a participatory mode integrating enriched compost (phospho-sulpho-nitro-compost) at 2 t ha^{-1}, substituting 5 FYM t ha^{-1} of the T_1 (INM_{FYM}), promoting on-farm residue recycling using wheat straw.
3. T_3 (STCR): Soil Test Crop Response inorganic input recommendations developed for the region through All India Coordinated Research Project on Soil Test Crop Response correlation (ICAR). Nutrient recommendations were calculated for each of the nine farm plots based on their initial soil test values to achieve specific target yields.
4. T_4 (FP): Farmers' practice involving 1 t ha^{-1} FYM with nitrogenous and phosphatic fertilizers.

The Recommended Dose of Fertilizer (RDF) used as the baseline for the INM treatments (T_1 and T_2) was: 20 kg ha^{-1} N, 60 kg ha^{-1} P_2O_5 and 20 kg ha^{-1} K_2O for soybean crop and 120 kg ha^{-1} N, 60 kg ha^{-1} P_2O_5 and 40 kg ha^{-1} K_2O for wheat crop. Biofertilizers were applied in dry

and liquid forms through seed treatments. The experiment followed a Randomized Block Design (RBD) in an on-farm setting, with each of the nine selected farmers serving as a replication. Treatment plots were large (1000 m² each), leading to a total evaluated area of 3.6 hectares.

Climatic parameters

Climatic factors influencing crop growth were monitored over the three-year study during the *Kharif* (June–July to October–November) and *Rabi* (October–November to March–April) seasons. Parameters such as maximum temperature, minimum temperature, average daily temperature and rainfall amount and distribution were monitored, particularly during the critical growth stages of these crops. These data were then subjected to correlation analysis with crop yields to evaluate the influence of weather conditions on overall productivity.

Soil analysis

Surface soil samples (0-15 cm depth) were collected pre- trial (before the first crop season)- and post-trial (after three years) from the nine farm fields. Initial composite soil samples were derived from five points per 4000m² area to established baseline conditions. The soil samples taken pre-trial from each treatment plot were analysed for the physical and chemical properties. Initial soil properties for the selected agroecosystem averaged as 45% sand, 13.5% silt and 41.5% clay content (Loam texture) with pH of 7.63, Electricity Conductivity (EC) of 0.2 ds m^{-1}, organic carbon - 6.3 g kg^{-1}, available nitrogen - 204.9 kg ha^{-1}, available phosphorus - 12.3 kg ha^{-1}, available potassium - 269 kg ha^{-1}. Specific nutrient recommendations for T_3 were based on these initial soil test values.

Intercultural operations

A cost-sharing approach was adopted to facilitate the field evaluation, with the study covering the cost of new inputs (seeds, chemical and biofertilizers, compost preparation materials (portable beds, rock phosphate, iron pyrite], and plant protection chemicals). Farmyard Manure (FYM) for T_1 and T_4 was sourced on-farm.

The soybean variety used was the early maturing JS 95-60 (average yield ~1.6 t ha^{-1}), and the wheat variety GW-322 (average yield ~4.5 t ha^{-1} even under late sowing). Seed rates were 80 kg ha^{-1} for soybean and 100 kg ha^{-1} for wheat for all treatments. Soybean sowing occurred mid-June to early July, contingent on monsoon onset, with wheat sowing 3-4 weeks post-soybean harvest in October. Wheat irrigation frequency varied based on water availability (3-5 irrigations). Soybean received basal NPK fertilizers; wheat received 50% basal nitrogen, full P and K, with remaining N top-dressed in two split doses along with irrigation. For the wheat crop, quantity and frequency of irrigations was based on water availability in the on-farm water resources, even though the crop received at least three irrigations in season. Herbicide (Imazethapyr 10% SL, 750 ml ha^{-1}) and pesticide (Triazophos 40 EC, 250 gm ha^{-1} AI) were applied to soybean crop as needed.

Total system productivity and economic analysis

Yields of grain and straw for both crops were recorded when crop plants attained physiological maturity. For yield estimation, one random 4 m^2 quadrat sample was collected from each treatment plot across the nine farms, and the resulting yields were extrapolated to kg ha^{-1}. The total system productivity of the soybean–wheat cropping system was evaluated by calculating the system yield. This involved converting the soybean yield into Wheat Equivalent Yield (WEY) using the respective annual market prices of the two crops. The wheat equivalent yield of soybean was then summed with the actual wheat yield to derive the overall system yield. An economic analysis was conducted to determine the gross returns and total production costs, deriving a benefit-cost ratio. Production costs accounted for machinery use, seeds, manures

and fertilizers, herbicides, and plant protection chemicals. Input prices and Minimum Support Prices (MSP) for soybean and wheat were tracked annually to assess farm-level economics. To evaluate the yield stability and consistency of the soybean–wheat system under different nutrient management practices, the Sustainable Yield Index (SYI) as proposed by Singh *et al.* (1990) was used.

$$SYI = (\bar{Y} - \sigma) / Y_{max}$$

Where: $\bar{\mathbf{Y}}$ = Mean yield over the years, $\boldsymbol{\sigma}$ = Standard deviation (a measure of risk or yield variability) and $\mathbf{Y_{max}}$ = Maximum observed yield. Here, low values of standard deviation and higher value of SYI indicates that the system is more sustainable.

Nutrient balance calculation

Nutrient balances were computed for major nutrients (NPK), considering inputs and outputs in kg ha^{-1} $year^{-1}$. Negative balances indicated nutrient depletion, while positive balances indicated accumulation. The average annual nutrient addition to the soybean-wheat system through manures and fertilizers under different nutrient management practices are given in Table 1.

Table 1. Average annual nutrient addition to soybean-wheat system under the four treatments

Treatments	Average nutrient addition in each crop season (kgha^{-1})						
	Organic manure				Inorganic fertilizers		
	N	P_2O_5	K_2O	S	N	P_2O_5	K_2O
**T_1** (INM$_{FYM}$)	25.0	10.0	25.0	0.0	100.0	75.0	40.0
**T_2** (INM$_{EC}$)	20.0	40.0	42.0	34.0	100.0	75.0	40.0
**T_3** (STCR)	0.0	0.0	0.0	0.0	156.3	188.0	91.6
**T_4** (FP)	5.0	2.0	5.0	0.0	100.0	145.0	0.0

Statistical analysis

Data collected over three crop seasons were subjected to Combined Analysis of Variance (ANOVA) appropriate to the experimental design for various agronomic and economic parameters. A pooled (combined) analysis was conducted for yield and economic indicators to evaluate treatment effects across years. Treatment means were compared using Tukey's Honest Significant Difference (HSD) test at a significance level of $P < 0.05$. All statistical analyses were performed using SAS software (Version 9.3), employing the Generalized Linear Model procedure (PROC GLM).

Results and Discussion

Climatic parameters

Climatic data recorded over the three years (Figure 1) indicated a distinct unimodal rainfall pattern characteristic of the region, with peak precipitation consistently occurring in July, ranging between 500 to 600 mm. This high-rainfall period (June to September) coincided with elevated maximum temperatures (30 to 36°C), during the *Kharif* season. Since the soybean variety used for the study was an early maturing one, duration of the crop was less than 100 days. Sowing was carried out after the effective onset of the monsoon (6–7 initial rainy days).

Fig 1. Climate Graph of all the three soybean-wheat crop rotation seasons during the study period

Temperature in the first *Kharif* season showed average seasonal temperature (T_{avg}) of 27°C with maximum (T_{max}) and minimum (T_{min}) monthly average of 33.6°C in June and 22.1°C in September, respectively. The monsoon starts in the region by the second week of June recorded 1473.0 mm total seasonal rainfall distributed over 76 rainy days. However, the second *Kharif*

season observed a slight change in the weather pattern due to the late monsoon arrival by the second week of July. The season recorded a T_{avg} value of 28.9°C, T_{max} of 33.1°C in the month of July and T_{min} of 19.2°C in the month of October. Moreover, the season received a weak rainfall of 858.7 mm distributed over 59 rainy days. Although the third *Kharif* season followed a similar temperature pattern as that of the first season with a T_{avg},T_{max}, T_{min} values of 27.7°C, 33.9°C, and 21.1°C respectively. The monsoon was not evenly distributed with a total seasonal rainfall of 1192.9 mm spread over a short period of 51 rainy days.

The graph shows that from October onward, both rainfall and temperatures began to decline in all the three seasons, with the *Rabi* season (October to April) characterized by minimal rainfall and cooler temperatures, especially in December and January when minimum temperatures dropped to 4– to 6°C. During the first *Rabi* season the average (T_{avg}) maximum (T_{max}) and minimum (T_{min}) were 22.3°C, 29.2°C (October), and 10.3°C (January), respectively. During the second season the same were recorded as 22.1°C, 29.5°C (November), and 8.3°C (January) 24.2°C, 31.9°C (November), and 10.6°C (December). These conditions are conducive to wheat growth, which typically benefits from dry weather and moderate temperatures during its vegetative and reproductive stages.

The southwest monsoon, which typically arrives in the Bhopal district by mid-June and continues until the end of September, plays a crucial role in ensuring water availability during the critical growth stages of the soybean crop, thereby improving the seed yield. Although the third crop season experienced substantial rainfall from June to August, a prolonged dry spell of 12 days in September coincided with the pod-filling stage of soybean. Drought stress during the pod development and filling stages can significantly reduce yield by decreasing the number of seeds per pod and resulting in smaller seed size (De Souza *et al*., 1997; Gebre and Earl, 2020; Zeleke and Nendel, 2024). Since groundwater availability for irrigation is influenced by seasonal precipitation patterns (Asoka and Mishra, 2020), variability in the arrival and

withdrawal of the monsoon—as well as the intensity and distribution of rainfall during the study period—may have adversely affected the growth and yield of rainfed soybean, and consequently the wheat crop, particularly on farms with limited on-farm water resources. Overall, the variability in the arrival and withdrawal of the monsoon, along with the intensity and distribution of rainfall during the study period, likely played a notable role in influencing the productivity of the rainfed soybean crop and, consequently, the succeeding wheat crop, particularly on farms with limited on-farm water resources. The overall climatic pattern during the study period supported the successful implementation of the soybean–wheat system.

Impact of different nutrient management practices on crop yield

The yield performance of soybean and wheat under different treatments during the three crop rotation seasons revealed significant effects of year, treatment, and their interaction on crop productivity and sustainability (Table 2).

Table 2. Average yield and Sustainable Yield Index (SYI) of soybean and wheat under different nutrient management practices in the soybean–wheat crop rotation system.

Crop	Treatments	Average Yield (t ha^{-1})				SYI
		Year 1	Year 2	Year 3	Pooled	
**SOYBEAN**	**T_1** (INM_{FYM})	1.14a	1.15a	0.91c	1.07b	0.82
	**T_2** (INM_{EC})	1.25a	1.22a	1.05a	1.17a	0.86
	**T_3** (STCR)	1.19a	1.17a	1.00b	1.12ab	0.85
	**T_4** (FP)	1.04a	0.98b	0.83d	0.95c	0.81
Sources of variation		*Probability level of significance*				
Year		-	-	-	*<0.0001*	

Treatment		*0.1345*	*0.0004*	*<0.0001*	*<0.0001*	
Year x Treatment		*-*	*-*	*-*	*<0.0001*	
**WHEAT**	$\mathbf{T_1}$ (INM$_{FYM}$)	4.06a	3.79b	3.90c	3.92b	0.93
	$\mathbf{T_2}$ (INM$_{EC}$)	4.39a	4.20a	4.25a	4.28a	0.95
	$\mathbf{T_3}$ (STCR)	4.13a	3.96b	4.08b	4.06b	0.96
	$\mathbf{T_4}$ (FP)	3.56b	3.42c	3.67d	3.55c	0.93
Sources of variation		*Probability level of significance*				
Year		*-*	*-*	*-*	*<0.0001*	
Treatment		*0.0003*	*<0.0001*	*<0.0001*	*<0.0001*	
Year x Treatment		*-*	*-*	*-*	*<0.0001*	

Means followed by the same letter/letters are not significantly different based on Tukey's Honest Significant Difference (HSD) at probability P=0.05.

For soybean, treatment T_2 (INM$_{EC}$) consistently recorded the highest yields across the three years, with a pooled average of 1.17 t ha^{-1} and the highest Sustainable Yield Index (SYI) of 0.86. T_3 (STCR) followed closely with a pooled yield of 1.12 t ha^{-1} and SYI of 0.85. Treatment T_4 (Farmers' Practice) recorded the lowest performance, with a pooled yield of 0.95 t ha^{-1} and the lowest SYI (0.81). Although treatment differences were not significant in the first year, they became statistically significant in subsequent years and in the pooled data, indicating a growing treatment impact over time. The significant Year × Treatment interaction ($p < 0.0001$) suggests that treatment effects varied considerably with climatic conditions, especially during the third year which recorded comparatively lower yields.

In wheat, treatment T_2 again emerged as the top performer with a pooled average yield of 4.28 t ha^{-1} and a high SYI of 0.95. T_3 also maintained strong performance with a pooled yield of 4.06 t ha^{-1} and the highest SYI among all treatments at 0.96, indicating strong yield stability

across years. T_4 had the lowest yield (3.55 t ha^{-1}), although it maintained a relatively high SYI of 0.93, indicating that while its productivity was lower, it was more consistent. Unlike soybean, treatment differences in wheat were significant in all individual years and the pooled analysis ($p < 0.0001$), highlighting a stronger response of wheat to treatment interventions. Again, the significant Year × Treatment interaction confirms that seasonal climatic conditions influenced treatment effectiveness.

Overall, the superior performance of the two nutrient management treatments T_2 (INM_{EC}) and T_3 (STCR) was consistent across both soybean and wheat in terms of yield and SYI. T_2 provided the highest overall productivity in both crops, while T_3 demonstrated the highest stability in wheat (SYI of 0.96). These findings underscore the importance of optimal nutrient management in maintaining yield stability under variable climatic conditions compared to the Farmers' Practice), as reflected in the SYI. These findings support the recommendation of T_2 as a preferred management strategy in the soybean–wheat system for achieving both productivity and resilience. Yield advantages resulting from the use of enriched compost (phospho-sulpho-nitro compost) for soybean and wheat crops have also been reported by Manna *et al*. (2001) and Pal *et al*. (2020).

Variation in crop yield across the farm holdings

The average seed yield of soybean and wheat crops from different nutrient management practices (Figure 2 and 3) showed notable variability across the farm holdings and years. As shown in Figure 2, treatment T_2 consistently produced the highest average soybean seed yields across most farm sizes in all three years, recording the highest yield of 1.6 t ha^{-1} in the first crop season. Soybean yields were generally higher in larger holdings (semi-medium), followed by medium and small holdings, suggesting a possible advantage of resource availability or management efficiency in larger farms. Treatment T_4 consistently recorded the lowest yields across all holding categories and years, highlighting its limited effectiveness. The first year

showed the most favourable yield performance overall, followed by second year, with a noticeable decline in the third year, likely influenced by climatic variations such as reduced rainfall or temperature extremes, as seen in the earlier climate graph.

For the wheat crop (Figure 3) the average grain yield across different farm holding sizes and treatments demonstrated consistent trends, with Treatment T_2 producing the highest yields across most categories. Semi-medium holdings consistently achieved the highest yields, peaking near or above 4.8 t ha^{-1}, indicating more efficient resource utilization and possibly better access to inputs or mechanization. Across all years, T_4 recorded the lowest yields, especially in small and medium holdings, reinforcing its relatively poor agronomic performance. Year-wise, the second crop season showed slightly superior overall grain yield compared to the other years, with T_2 maintaining a lead regardless of farm size. Notably, the difference in yields among holding types narrowed slightly in the third year, perhaps reflecting uniform environmental stress or improved management practices across holdings.

The error bars across categories in both graphs indicate a moderate variability, with greater consistency in both soybean and wheat crop yields from $T_{2,}$ developed through a participatory technology development approach. These trends emphasize that T_2 is the most effective treatment for enhancing yield from the soybean-wheat rotation system irrespective of holding size likely due to the better adaptability to recommended practices. The semi-medium holdings consistently outperform smaller categories, possibly due to better input access and agronomic practices.

Fig 2. Yield of soybean across farm holdings from different nutrient management options during the study period

Fig 3. Yield of wheat across farm holdings from different nutrient management options during the study period

Economic performance of the soybean-wheat rotation system

An integrated assessment of yield, cost of cultivation, gross and net income, and benefit-cost ratio (BCR) across four treatments in the soybean–wheat system reveals significant difference between treatment on both crop productivity and economic returns over the three-year period (Table 3).

Table 3. Economic aspects of soybean, wheat and soybean-wheat system (average of 3 years)

Crop	Treatments	System Yield (Pooled)	Cultivation Cost (**INR ha^{-1})	Gross Income (INR ha^{-1})	Net Income (INR ha^{-1})	BCR
**SOYBEAN**	**T_1** (INM$_{FYM}$)	2.14b*	18603.00b	35236.67b	16633.67b	1.9c
	**T_2** (INM$_{EC}$)	2.35a*	20203.00a	38756.67a	18553.67b	1.9c
	**T_3** (STCR)	2.25ab*	14650.11c	37082.22ab	22432.11a	2.6a
	**T_4** (FP)	1.88c*	14750.00c	31313.33c	16563.33b	2.1b
Sources of variation			*Probability level of significance*			
Year		*<0.0001*	*<0.0001*	*<0.0001*	*<0.0001*	*<0.0001*
Treatment		*<0.0001*	*<0.0001*	*<0.0001*	*<0.0001*	*<0.0001*
Year x Treatment		*<0.0001*	*<0.0001*	*<0.0001*	*<0.0001*	*<0.0001*
**WHEAT**	**T_1** (INM$_{FYM}$)	3.92b	19300.00c	62666.67b	43366.67b	3.3b
	**T_2** (INM$_{EC}$)	4.28a	19300.00c	68426.67a	49126.67a	3.5a

	**T_3** (STCR)	4.06b	25347.67a	64911.41b	39563.74c	2.6c
	**T_4** (FP)	3.55c	21750.00b	56770.37c	35020.37d	2.6c
Sources of variation		*Probability level of significance*				
Year		*<0.0001*	*0.0035*	*0.0022*	*0.0056*	*0.0122*
Treatment		*<0.0001*	*<0.0001*	*<0.0001*	*<0.0001*	*<0.0001*
Year x Treatment		*<0.0001*	*<0.0001*	*<0.0001*	*<0.0001*	*<0.0001*
**SOYBEAN-WHEAT SYSTEM**	**T_1** (INM_{FYM})	6.05b	37903.00b	97903.33c	60000.33b	2.6ab
	**T_2** (INM_{EC})	6.63a	39503.00a	107183.33a	67680.33a	2.7a
	**T_3** (STCR)	6.30ab	39997.78a	101993.63b	61995.85b	2.6b
	**T_4** (FP)	5.43c	36500.00c	88083.70d	51583.70c	2.4c
Sources of variation		*Probability level of significance*				
Year		*<0.0001*	*<0.0001*	*<0.0001*	*<0.0001*	*<0.0001*
Treatment		*<0.0001*	*<0.0001*	*<0.0001*	*<0.0001*	*<0.0001*
Year x Treatment		*<0.0001*	*<0.0001*	*<0.0001*	*<0.0001*	*<0.0001*

Means followed by the same letter/letters are not significantly different based on Tukey's Honest Significant Difference (HSD) at probability P=0.05.

*** Wheat equivalent yield** **** 1 INR = 0.012 USD = 0.010 EUR**

In the case of soybean, the pooled average seed yield of three years was highest for T_2 (1.17 t ha^{-1}), followed by T_3 (1.12 t ha^{-1}), with T4 recording the lowest (0.95 t ha^{-1}). Although T_2 recorded the highest gross income (38,756.67 INR ha^{-1}), its net income (18,553.67 INR ha^{-1}) was lower than T_3 due to additional cost of off-farm inputs for the preparation of enriched compost. T_3 emerged as the most economically efficient nutrient management practice for

soybean, with the highest net income (22,432.11 INR ha^{-1}) and a superior Benefit Cost Ratio (BCR) of 2.6, owing to its relatively low cost of cultivation because of soil test-based fertilizer application. T_4 despite having a low yield, maintained a moderate BCR of 2.1 due to its low cultivation cost.

For the wheat crop, T_2 again demonstrated the highest yield (4.28 t ha^{-1}) and generated the maximum net income (49,126.67 INR ha^{-1}) with the highest BCR (3.5), highlighting its economic superiority. T_3, while yielding moderately (4.06 t ha^{-1}), was associated with a high cultivation cost (25,347.67 INR ha^{-1}) due to the more fertilizer requirement, which resulted in a reduced net income to 39,563.74 INR ha^{-1} and BCR to 2.6. T_4 had the lowest yield (3.55 t ha^{-1}) and net income (35,020.37 INR ha^{-1}), and its BCR also remained at 2.6. This indicates that while T_3 was cost-intensive, its returns did not proportionally increase.

Considering the soybean–wheat rotation system, T_2 was the best performer with 6.63 t ha^{-1} in terms of the overall system yield, followed by T_3 (6.30 t ha^{-1}), with T_4 recording the lowest at 5.43 t ha^{-1}. The highest gross (107,183.33 INR ha^{-1}) and net income (67,680.33 INR ha^{-1}) were also associated with T_2. T_3 maintained competitive performance in both productivity and profitability, while T_1 showed moderate results. Although T_4 had the lowest productivity and income, it achieved a reasonable BCR of 2.4, indicating some economic viability due to lower inputs. Furthermore, the statistical analysis confirmed that year, treatment, and year × treatment interactions significantly influenced all economic and yield parameters ($p < 0.0001$). This suggests that the year-to-year variations in rainfall intensity and distribution, particularly during the monsoon months, likely contributed to the significant differences observed in system yields and economic returns across treatments.

Impact of nutrient management practices on soil health

Nutrient budgeting of different nutrient management systems

The nutrient balance analysis for nitrogen (N), phosphorus (P), and potassium (K) under the soybean–wheat cropping system (Table 4) revealed significant differences across the four treatments. Treatment T_3 recorded the most balanced nutrient dynamics, with relatively lower negative balances for nitrogen (–19.2 kg ha^{-1}) and potassium (–19.3 kg ha^{-1}), along with the highest positive phosphorus balance (+52.9 kg ha^{-1}), indicating efficient nutrient utilization and minimal losses. In stark contrast, treatment T_4 showed the most severe nutrient depletion, particularly for potassium (–104.3 kg ha^{-1}) and nitrogen (–52.5 kg ha^{-1}), likely due to inadequate potassium application (only 4 kg ha^{-1}). However, T_4 exhibited a higher phosphorus uptake than input, it still maintained a small positive P balance.

Treatment T_2, despite receiving higher nutrient inputs, recorded substantial deficits in N (–42.1 kg ha^{-1}) and K (–42.7 kg ha^{-1}), suggesting either poor nutrient uptake efficiency or higher crop demand, both of which may lead to long-term soil fertility degradation if not addressed. Treatment T_1 showed moderate performance, with deficits in N (–17.7 kg ha^{-1}) and K (–57.0 kg ha^{-1}), and a modest P surplus (+19.4 kg ha^{-1}).

Table 4. Nutrient balance sheet of soybean-wheat crop rotation system under different nutrient management practices

Treatments	Nutrient applied to soil (kg ha^{-1})			Nutrient removal by crops (kg ha^{-1})			Apparent Nutrient Balance (kg ha^{-1})		
	N	P	K	N	P	K	N	P	K
**T_1** (INM$_{FYM}$)	145	46	59	162.7	26.4	115.9	-17.7	19.4	-57.0
**T_2** (INM$_{EC}$)	140	59	80	182.1	28.2	122.7	-42.1	30.7	-42.7
**T_3** (STCR)	148	80	86	167.2	27.1	105.3	-19.2	52.9	-19.3
**T_4** (FP)	97	51	4	149.5	31.7	108.3	-52.5	19.3	-104.3

These findings reinforce that balanced nutrient application, as demonstrated in T_3, promotes nutrient retention and system sustainability, while imbalanced or insufficient fertilization, as in T_4, accelerates nutrient mining and threatens soil health. Similar concerns over nutrient mining in Indian soils have been reported by Surendran *et al*. (2016), who advocate for integrated nutrient management strategies. Furthermore, Jayanthi *et al*. (2024) emphasized improved nutrient uptake efficiency under soil test–based nutrient application, supporting the observed benefits in T_3.

Effect of nutrient management practices on soil microbial populations

The average on soil microbial populations (Table 5) reveals significant variations in the abundance of bacteria, fungi, and actinobacteria across the different nutrient management treatments. Bacterial populations (expressed as $\times10^7$ cfu g^{-1} dry soil) were highest under Treatment T_3 (2.39), which was significantly greater than T_2 and statistically comparable to T_1, indicating enhanced bacterial activity potentially due to more balanced nutrient availability in T_3.

In contrast, fungal populations ($\times10^4$ cfu g^{-1} dry soil) peaked in Treatment T_2 (3.46), showing a statistically significant increase over all the other treatments. This suggests that the nutrient regime in T_2 was particularly favorable for fungal proliferation, possibly due to specific organic inputs or microenvironmental conditions.

Table 5. Effect of different treatments on Soil microbial populations

Treatments	Bacteria<br>X10^7 cfu g^{-1} dry soil	Fungi	Actinobacteria

		**X10^{4} cfu g^{-1} dry soil**	**X10^{5} cfu g^{-1} dry soil**
**T_1** (INM_{FYM})	1.91ab	2.63b	3.01b
**T_2** (INM_{EC})	1.34b	3.46a	3.50a
**T_3** (STCR)	2.39a	2.09b	2.84b
**T_4** (FP)	1.45ab	1.30c	2.39c
Sources of variation	*Probability level of significance*		
Treatment	*0.0326*	*<0.0001*	*<0.0001*

Means followed by the same letter/letters are not significantly different based on Tukey's Honest Significant Difference (HSD) at probability P=0.05.

Actinobacterial counts, which play a key role in organic matter decomposition and disease suppression, were highest in T_2 (3.50), followed by T_1 and T_3. Treatment T_4 consistently recorded the lowest microbial counts across all groups—fungi (1.30), bacteria, and actinobacteria (2.39)—indicating a relatively less biologically active soil environment, possibly due to imbalanced nutrient inputs or suboptimal soil conditions.

The statistically significant treatment effects ($p < 0.05$) observed across microbial groups underscore the influence of nutrient management on the soil microbial ecology. Notably, T_2 enhanced both fungal and actinobacterial populations, while T_3 was more effective in promoting bacterial abundance. These findings highlight the potential of tailored nutrient strategies to improve soil biological activity and overall soil health in the soybean–wheat cropping system.

The importance of nutrient inputs—both organic and inorganic—in modulating soil microbial diversity and function has also been emphasized in earlier studies by Geisseler and Scow (2014), Zhou *et al*. (2015), and Yadav *et al*. (2018).

Conclusion

This study examined the impact of different nutrient management practices on productivity, profitability, and soil health under the soybean–wheat rotation system of Central India, amid increasing climatic variability. The findings clearly demonstrate that nutrient management plays a pivotal role in sustaining yield and income, particularly under rainfed agriculture. Among the tested treatments, T_2—which combined enriched compost, biofertilizers, and chemical fertilizers—consistently delivered the highest system yield (6.63 t ha^{-1}), net income (67,680.33 INR ha^{-1}), and benefit-cost ratio (3.5) while maintaining a high sustainable yield index. This treatment also significantly enhanced fungal and actinobacterial populations, indicating improved soil biological activity. T_3, with soil test–based nutrient application, emerged as the most nutrient-balanced system, promoting bacterial abundance and offering competitive economic and yield performance. T_2 and T_3 proved more adaptable across diverse climatic scenarios and farm sizes, with semi-medium holdings achieving the best outcomes due to better input access.

Overall, the current research study emphasized on importance of integrated and balanced nutrient management in enhancing crop productivity, economic viability, and long-term soil health in the soybean–wheat system. The participatory and site-specific approaches adopted in T_2 and T_3 present scalable options for resource-limited farmers to adapt to climate change while ensuring sustainable agricultural intensification. Future research should focus on long-term nutrient cycling, water-use efficiency, and expanding these strategies to other agro-ecological regions to enhance system resilience.

Acknowledgments

The authors sincerely acknowledge the support of Indian Council of Agricultural Research to carry out this research. The cooperation of farmers of the study area is also gratefully appreciated.

References

1. Asoka A, Mishra V. A strong linkage between seasonal crop growth and groundwater storage variability in India. J. Hydrometeorol. 2020; 22,125–138. https://doi.org/10.1175/JHM-D-20-0085.1.
2. Behera U K, Sharma A R, Pandey H N. Sustaining productivity of wheat-soybean cropping system through integrated nutrient management practices on the vertisols of central India. Plant and Soil 2007; 297:185-199. doi.org/10.1007/s11104-007-9332-3.
3. Caviglia O P, Sadras V O, Andrade F H. Yield and quality of wheat and soybean in sole and double cropping. Agron. J. 2011; 103(4), 1081-1089. doi.org/10.2134/agronj2011.0019.
4. De Souza P I, Egli D B, Bruening W P. Water stress during seed filling and leaf senescence in soybean. Agron. J. 1997; 89, 807-812. https://doi.org/10.2134/agronj1997.00021962008900050015x.
5. Dubey M, Shinogi K C, Awasthi S K, Khan MA. Comparing the nutrient management pattern in soybean and rice based cropping systems by soil health card holders and non-holders. Indian J. Ext. Educ. 2022; 58(3), 83-87. https://doi.org/10.48165/IJEE.2022.58318.

6. Dupare B U, Sharama P, Verma S K, Billore S D. Adoption of soybean production technology by the farmers in Malwa plateau of Madhya Pradesh. Soybean Research 2019; 17(1&2), 62-76.

7. Gebre M H, Earl H J. Effects of Growth Medium and Water Stress on Soybean [*Glycine max* (L.) Merr.] Growth, Soil Water Extraction and Rooting Profiles by Depth in 1-m Rooting Columns. Front. Plant. Sci. 2020; 11, 487. https://doi.org/10.3389/fpls.2020.00487.

8. Geisseler D, Scow K M. Long-term effects of mineral fertilizers on soil microorganisms–A review. Soil. Biol. Biochem. 2014; 75, 54–63. https://doi.org/10.1016/j.soilbio.2014.03.023

9. Government of India. All India Report on Agriculture Census 2015-16., Department of Agriculture & Farmers Welfare. 2020. https://agcensus1.da.gov.in/tehsilsummarytype.aspx.

10. Government of India. Agricultural Statistics at a Glance 2022. Ministry of Agriculture & Farmers welfare. 2023. https://agriwelfare.gov.in/Documents/CWWGDATA/Agricultural_Statistics_at_a_Glance_2022_0.pdf.

11. ICAR-IISR. Director's Report of AICRP on Soybean 2020-21. ICAR-Indian Institute of Soybean Research, Indore, Madhya Pradesh, India. 2021. https://iisrindore.icar.gov.in/pdfdoc/AicrpsAR2020-21.pdf.

12. Jain N, Bhatia A, Pathak H. Emission of air pollutants from crop residue burning in India. Aerosol Air Qual. Res. 2014; 14, 422-430. https://doi.org/10.4209/aaqr.2013.01.0031.

13. Jayanthi T, Latha H S, Madappa S, Umilsingh N, Umesh H R, Ashok LB. Budgeting of primary nutrients in soil under rice-cowpea cropping sequences in Alfisols. J. Adv.

Biol. Biotechnol. 2024; 27 (10), 1046-61. https://doi.org/10.9734/jabb/2024/v27i101527

14. Kumar P, Kumar S, Joshi L. Socioeconomic and Environmental Implications of Agricultural Residue Burning. Springer Briefs in Environmental Science. Springer, New Delhi. 2015. https://link.springer.com/chapter/10.1007/978-81-322-2014-5_2.

15. Kundu S, Bhattacharyya R, Prakash V, Gupta H S, Pathak H. Ladha J K. Long term yield trend and sustainability of rainfed soybean-wheat system through farmyard manure application in a sandy loam soil of the Indian Himalayas. Biol. Fertil. Soils. 2007; 43, 271-280. https://link.springer.com/article/10.1007/s00374-006-0102-9.

16. Kushwah S S, Reddy D D, Somasundaram J, Srivastava S, Khamparia R S. Crop residue retention and nutrient management practices on stratification of phosphorus and soil organic carbon in the soybean–wheat system in vertisols of Central India. Commun. Soil. Sci. Plant. Anal. 2016; 47, 2387-2395. https://www.tandfonline.com/doi/full/10.1080/00103624.2016.1243703.

17. Leff B, Ramankutty N, Foley J A. Geographic distribution of major crops across the world, Global Biogeochem. Cycles. 2004; 18, 1-27. doi.org/10.1029/2003GB002108.

18. Lu H, Hu L, Zheng W, Yao S, Qian L. Impact of household land endowment and environmental cognition on the willingness to implement straw incorporation in China. J. Clean. Prod. 2020; 262, 121479. https://doi.org/10.1016/j.jclepro.2020.121479.

19. Manna M C, Ghosh P K, Ghosh B N, Singh K N. Comparative effectiveness of phosphate-enriched compost and single superphosphate on yield, uptake of nutrients and soil quality under soybean - Wheat rotation. J. Agric. Sci. 2001; 137, 45-54. https://doi.org/10.1017/S0021859601008942.

20. Meena B P, Biswas A K, Singh M, Chaudhary R S, Singh A B, Das H, et.al. Long-term sustaining crop productivity and soil health in maize–chickpea system through integrated nutrient management practices in Vertisols of central India. Field. Crops. Res. 2019; 232, 62-76. https://www.sciencedirect.com/science/article/pii/S0378429018314096.

21. Morrison M J, Cober E R, Gregorich E, Voldeng H D, Ma B, Topp G C. Tillage and crop rotation effects on the yield of corn, soybean and wheat in eastern Canada. Can. J. Plant. Sci. 2018; 98(1), 183-191. doi/10.1139/CJPS-2016-0407.

22. Pal A, Deshmukh P W, Chaure P R, Bhoyar S M. Assessing comparative effectiveness of phosphocompost and nitro-phospho-sulpho compost on yield, nutrient uptake of soybean crop and soil fertility on Vertisols. Journal of Pharmacognosy and Phytochemistry 2020; 9(3), 2272-2276. https://www.phytojournal.com/archives/2020/vol9issue3/PartAK/9-3-367-639.pdf.

23. Reddy D D, Kushwaha S, Srivastava S, Khamparia RS. Long-term wheat residue management and supplementary nutrient input effects on phosphorus fractions and adsorption behavior in a vertisol. Commun. Soil. Sci. Plant. Anal. 2014; 45(4), 541-554. https://doi.org/10.1080/00103624.2013.867043.

24. Reddy K S, Mohanty M, Rao D L N, Subba Rao A, Pandey M, Singh M, et. al. Farmer involvement in the development and adoption of improved nutrient management technologies using the mother–baby trial approach in vertisols. Proc. Natl. Acad. Sci. India Sect. B Biol. Sci. 2015; 85, 51-62. https://doi.org/10.1007/s40011-013-0243-1.

25. Sattler C, Nagel U J. Factors affecting farmers' acceptance of conservation measures-A case study from north-eastern Germany. Land Use Policy. 2010; 27, 70-77. https://doi.org/10.1016/j.landusepol.2008.02.002.

26. Sharma P. Costs, returns and profitability of soybean cultivation in India: trends and prospects. Economic Affairs. 2016; 61(3), 413-425. DOI:10.5958/0976-4666.2016.00052.8

27. Singh R P, Das S K, Rao U M B, Reddy M N. Towards Sustainable Dryland Agricultural Practices. Bulletin 1990; 5–9. Central Research Institute for Dryland Agriculture, Hyderabad

28. Srivastava S, Rao A S, Biswas A K. Results of the participatory diagnosis of constraints and opportunities (PDCO) based trials from the state of Madhya Pradesh, in: Rao A S, Srivastava S (Eds.), Development of Farmers' Resource based Integrated Plant Nutrient Supply Systems: Experiments of a FAO-ICAR-IFFCO Collaborative Project and AICRP on Soil Test Crop Response Correlation, Indian Institute of Soil Science, Bhopal, Madhya Pradesh, India. 2001; 10-49.

29. Surendran U, Subramoniam S R, Raja P, Kumar V. Murugappan V. Budgeting of major nutrients and the mitigation options for nutrient mining in semi-arid tropical agro-ecosystem of Tamil Nadu, India using NUTMON model. Environ. Monit. Assess. 2016; 188, 250. https://doi.org/10.1007/s10661-016-5202-x.

30. United States Department of Agriculture. World Agricultural Production, Foreign Agricultural Service Circular Series, WAP 6-24 June 2024. https://apps.fas.usda.gov/psdonline/circulars/production.pdf.

31. Watson C A, Reckling M, Preissel S, Bachinger J, Bergkvist G, Kuhlman T, et. al. Grain legume production and use in European agricultural systems. Adv. Agron. 2017;144, 235–303. doi.org/10.1016/bs.agron.2017.03.003.

32. Yadav A N, Verma P, Kumar S, Sangwan P, Mishra S, Panjiar N, Saxena AK. Plant microbiomes and sustainable agriculture: Diversity and beneficial interactions. Microbiol. Res. 2018; 206, 131–143. https://doi.org/10.1016/j.micres.2017.08.001.

33. Yigezu Y A, El-Shater T, Boughlala M, Bishaw Z, Niane A A, Maalouf F, et. al. Legume-based rotations have clear economic advantages over cereal monocropping in dry areas. Agron. Sustain. Dev. 2019; 39, 58. https://doi.org/10.1007/s13593-019-0602-2.
34. Zeleke K, Nendel C. Yield response and water productivity of soybean (Glycine max L.) to deficit irrigation and sowing time in south-eastern Australia. Agric. Water. Mang. 2024; 296: 108815. https://doi.org/10.1016/j.agwat.2024.108815.
35. Zhou J, Guan D, Zhou B, Zhao B, Ma M, Qin J, et. al. Influence of 34-years of fertilization on bacterial communities in an intensively cultivated black soil in northeast China. Soil. Biol. Biochem. 2015; 90, 42–51. https://doi.org/10.1016/j.soilbio.2015.07.005.
36. Zuo Y, Zhang F. Iron and zinc biofortification strategies in dicot plants by intercropping with gramineous species: A review. Sustain. Agric. 2009; 29, 571–582. https://doi.org/10.1051/agro:2008055.

Figure

Click here to access/download;Figure;Figures.docx

FIGURES

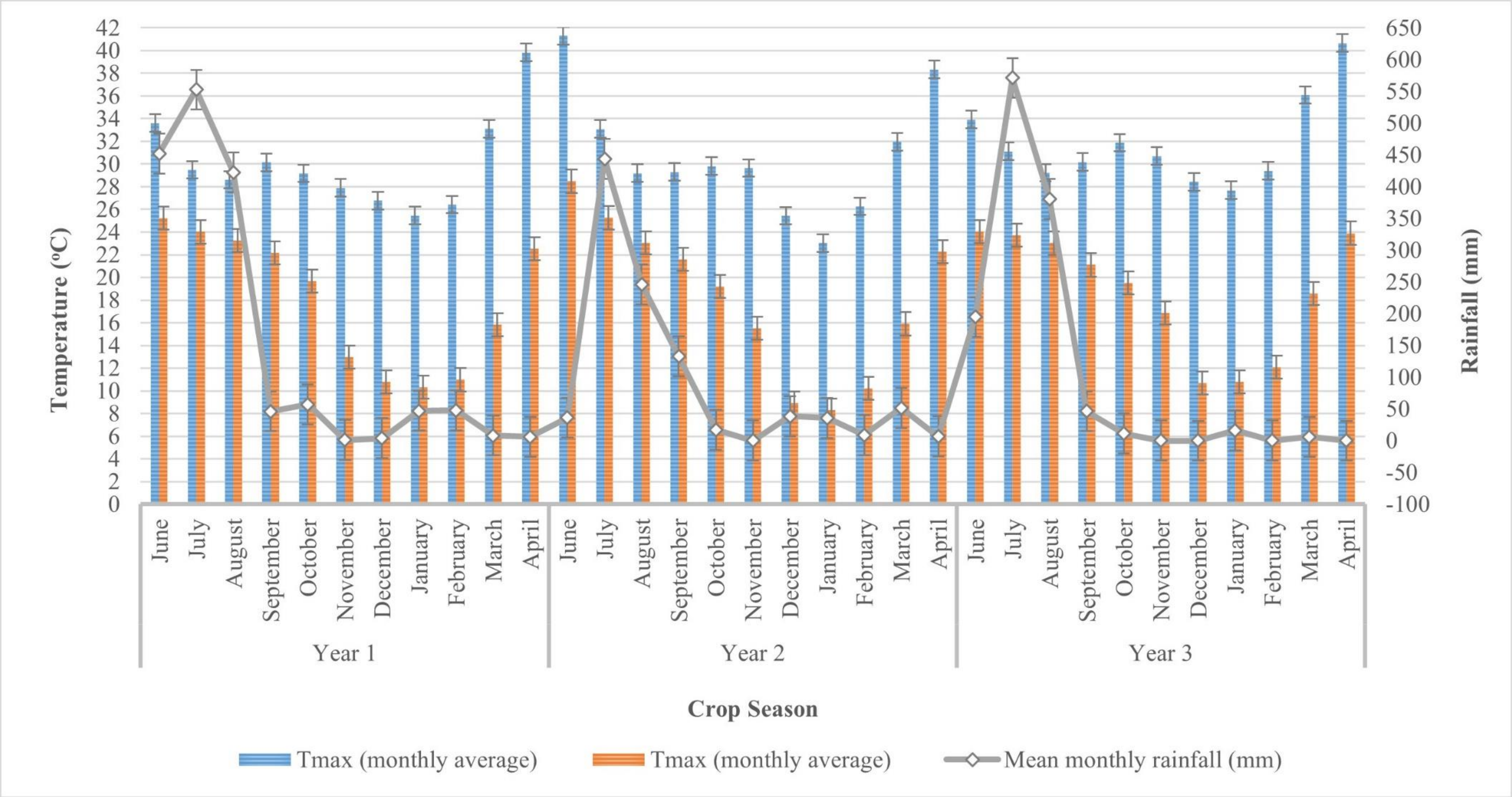

Fig 1. Climate Graph of all the three soybean-wheat crop rotation seasons during the study period

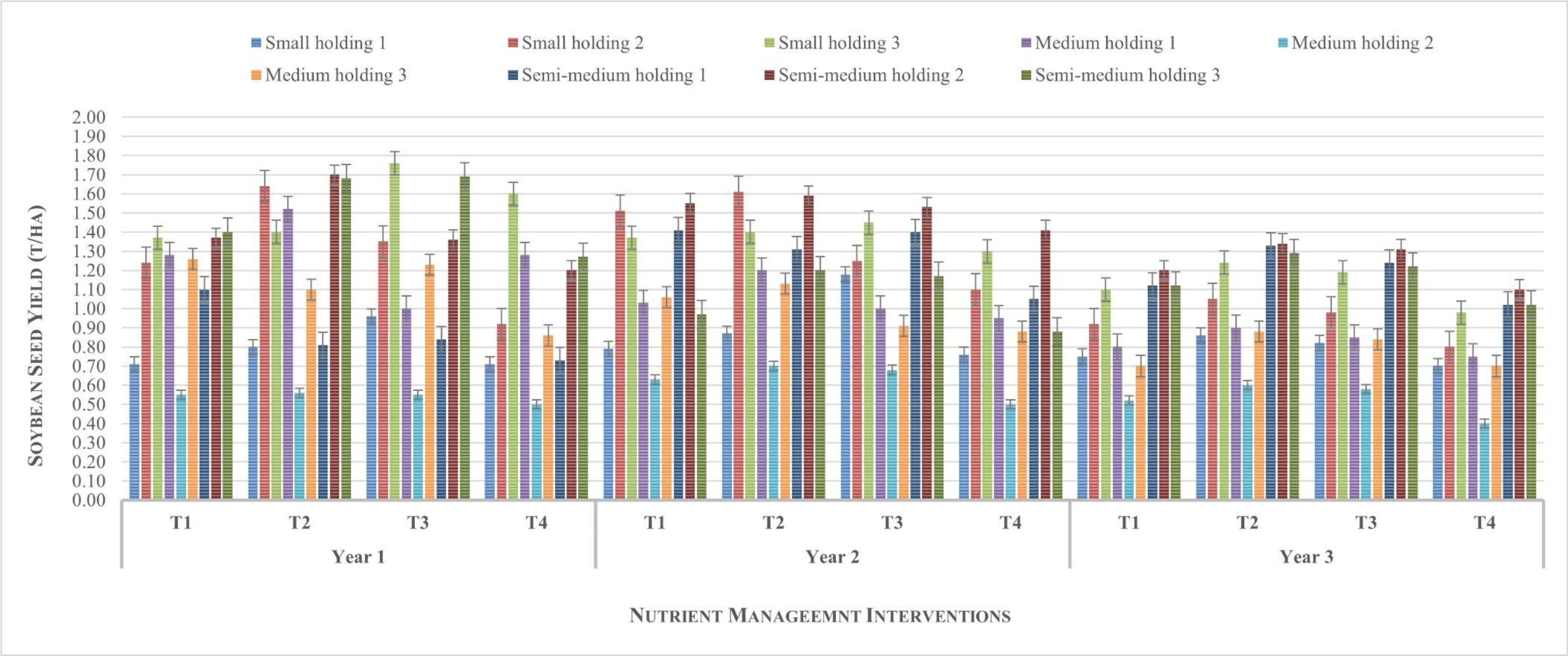

Fig 2. Yield of soybean crop across farm holdings from different nutrient management options during the study period

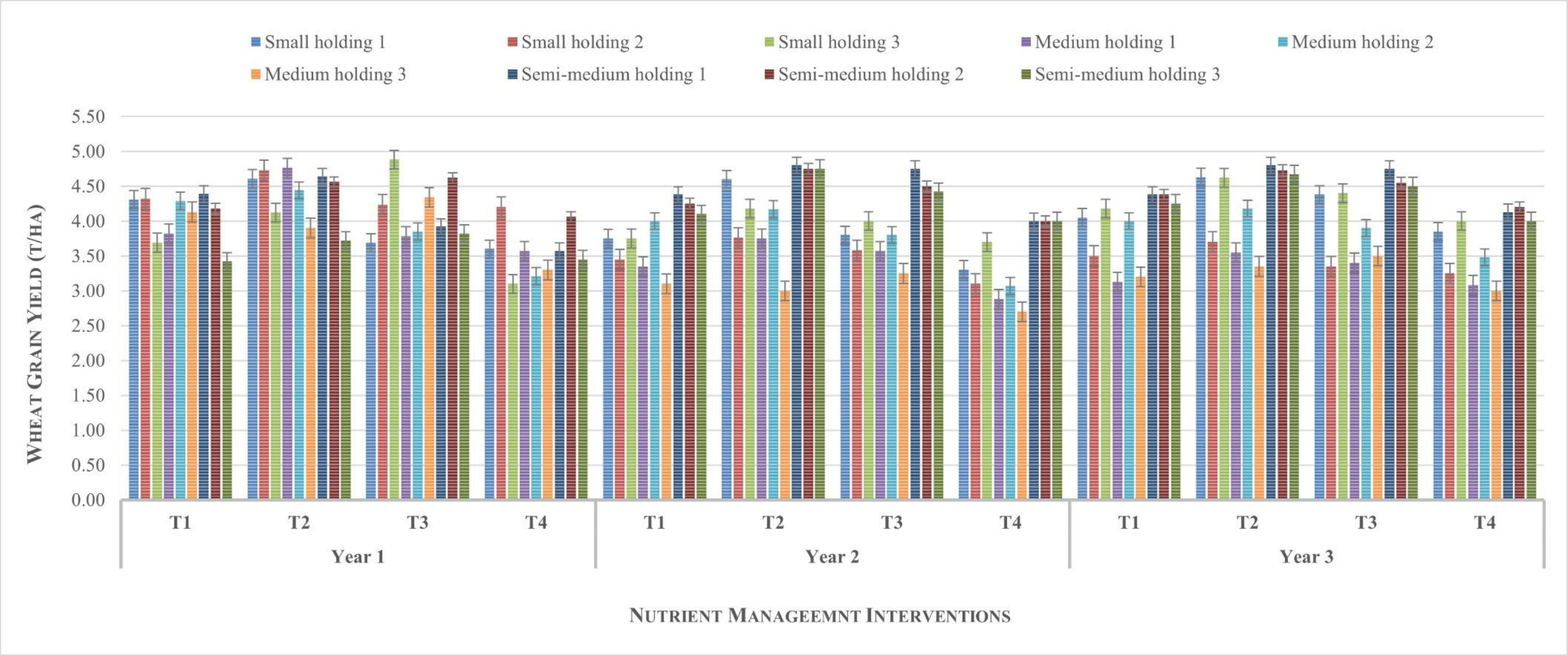

Fig 3 Yield of wheat crop across farm holdings from different nutrient management options during the study period

Click here to access/download
Supporting Information
Supporting Data.xlsx

TITLE, AUTHOR, AFFILIATION

Soil fertility management for yield and income stability in soybean-wheat rotation system under climate variability in subtropical Central India

Shinogi K C[1], Sanjay Srivastava[1*], Radha T K[2], D L N Rao[1#], Bharat Prakash Meena[1], Nishant Kumar Sinha[1], Hiranmoy Das[3], Rashmi I[4], Rosin K G[5], A B Singh[1#], Sanjib Kumar Behera[1], Monoranjan Mohanty[1]

Author Names and Affiliations:

[1] ICAR-Indian Institute of Soil Science, Bhopal, Madhya Pradesh, India

[2]ICAR-Indian Institute of Horticultural Research, Bangaluru, Karnataka, India

[3]ICAR-Indian Institute of Vegetable Research, Varanasi, Uttar Pradesh

[4]ICAR-Indian Institute of Soil and Water Conservation RC Kota, Rajasthan, India

[5] ICAR-Indian Agricultural research Institute, New Delhi, India

[#] Retired

* Corresponding Author:

E-mail: sanjaysrivastava238@gmail.com

Abstract

The soybean–wheat rotation system forms a crucial ~~part~~ component of Central India's rainfed agriculture. Although historically productive and profitable, ~~While traditionally known for its productivity and profitability~~, the system's sustainability is increasingly threatened by suboptimal nutrient management, and soil degradation under changing climate. ~~To examine how nutrient management practices and farmers' efficiency in utilizing on-farm resources influence yield and income in soybean–wheat rotation system under variable environmental conditions in India a three-year study was conducted in Central India using a Participatory Technology Development (PTD) approach~~. This three-year study, conducted on small and semi-medium farms in Central India using a Participatory Technology Development (PTD) approach, aimed to evaluate how diverse nutrient management strategies influence yield, profitability, and resource use efficiency. Four nutrient management strategies were tested on small to semi-medium farms: (T_1) I~~i~~ntegrated N~~n~~utrient M~~m~~anagement (INM) using chemical fertilizers, farmyard manure and biofertilizers, (T_2) I~~i~~ntegrated ~~n~~Nutrient M~~m~~anagement using chemical fertilizers, enriched compost and biofertilizers, (T_3) S~~s~~oil test crop response-based fertilization, and (T_4) F~~f~~armers' conventional practice. Among the four treatments T_2 achieved the highest system productivity (6.63 t ha^{-1} wheat equivalent), net returns (67,680 INR ha^{-1}), and superior yield stability, under climatic variability. T_3 offered moderate productivity while promoting ~~with~~ balanced nutrient use and lower input costs. Both T_2 and T_3 enhanced microbial activity and soil health, while T_4 led to nutrient depletion, (especially potassium), and low returns. Furthermore, ~~It is noted that~~ the timing and distribution of monsoon rainfall significantly impacted performance and yield stability of soybean. This study highlights the critical need for integrated, site-specific, and farmer-centric nutrient management strategies to

Formatted: Font: Not Bold

ensure sustainable productivity, profitability, and soil health under changing climatic conditions.

Introduction

Crop diversification through legume–cereal rotation is an effective soil fertility management technique practiced globally. ~~widely practiced across the world~~. As a low-cost and sustainable strategy, this ~~legume–cereal~~ rotation supports both ecological intensification and economic resilience in diverse agroecosystems, particularly for smallholder and rainfed agriculture systems (Yigezu *et al*., 2019; Meena *et al*., 2019). Legumes~~inous crops~~ contribute nitrogen (N)-rich residues with a low carbon-to-nitrogen (~~C:N~~C: N) ratio, which significantly enhances the yield of subsequent cereal crops (Watson *et al*., 2017; Morrison *et al*., 2018). Additionally, alternating legumes with cereals is regarded as a natural method of biofortification, improving the nutritional value of cereal grains (Zuo and Zhang, 2009).

Globally, ~~L~~legume–cereal rotation systems exhibit wide global diversity, reflecting variations in land suitability and the climatic adaptability of different legumes and cereals. According to Leff *et al*. (2004), wheat (*Triticum aestivum*) is the most widely cultivated cereal, while soybean (*Glycine max*) is the leading leguminous oilseed crop globally. The soybean–wheat rotation system is especially predominant in temperate and subtropical regions (Caviglia *et al*., 2011), owing to its high productivity, profitability, and compatibility with regional climate conditions.

In India, the soybean–wheat rotation system has been practiced on the black soils (Vertisols) of Central India since the early 1980s, following the introduction of soybean as a rainfed crop ~~into the traditional fallow–wheat system~~ in Madhya Pradesh. This rotation system was initially highly profitable, contributing significantly to regional agricultural incomes (Srivastava *et al*., 2001; Behera *et al*., 2007; Reddy *et al*., 2015). However, in recent years, climate change has severely disrupted its stability. Increasingly erratic rainfall patterns, drought stress during

Formatted: Font: Italic
Formatted: Font: Italic
Formatted: Font: Italic
Formatted: Font: Italic
Formatted: Font: Italic
Formatted: Font: Italic
Formatted: Font: Italic
Formatted: Font: Italic

critical reproductive stages, and the emergence of new pests and diseases have severely impacted soybean yields. The national average soybean yield has declined to around 1.0 t ha^{-1}, which is significantly below the global average of 2.76 t ha^{-1} (GoI, 2023; USDA, 2024).

Concurrently with Alongside climate change, adoption of higher seed rates than recommended and unscientific nutrient management practices have further reduced the profitability of soybean-wheat rotation system in India, despite wheat maintaining higher yields (Sharma, 2016). Furthermore, in-situ burning of mechanically harvested wheat residues before summer ploughing has linked the soybean-wheat rotation system to environmental degradation. This practice deteriorates the soil by depleting organic matter and nutrients present in crop residues and destroying beneficial soil microorganisms (Jain *et al*., 2014; Reddy *et al*., 2014; Kumar *et al*., 2015).

To enhance the sustainability of make the soybean-wheat rotation system sustainable in India, a range of agricultural technologies have been integrated over the years, particularly to build system resilience by improving soil health to withstand biotic and abiotic stresses, and by promoting stress-tolerant crop varieties. In line with the findings of long-term fertilizer experiments by Kundu *et al.* (2007) in the soybean-wheat system, soil researchers have developed precise nutrient management practices. These include integrating organic and inorganic fertilizers, retention of crop stubble in the field either as soil cover or through soil incorporation, and the recycling of stubble into nutrient-rich composts (Reddy *et al*., 2014; Reddy *et al*., 2015). Additionally, to facilitate minimal soil disturbance during soybean sowing while retaining wheat stubble, customized machinery has been introduced to farmers (Kushwah *et al*., 2016).

However, dDespite substantial efforts by researchers and government agencies, studies have shown that field-level adoption of many of the recommended practices for soybean-based

Formatted: Font: Italic

Formatted: Font: Italic

Formatted: Font: Italic

Formatted: Font: Italic

Formatted: Font: Italic

Formatted: Font: Italic

Formatted: Font: Italic

systems remains insufficient to achieve their intended ecological and economic benefits (Dupare *et al*., 2019; Dubey *et al*., 2022).

Consequently, ~~S~~social researchers note that farmers' adoption decisions are not solely driven by economic profitability. Instead, they are significantly influenced by factors such as the time, labour, and effort required to implement the practices, as well as the perceived practicality and utility of the technologies within the specific context of individual farming systems. For example, in the case of crop residue incorporation, a farmer's environmental awareness and willingness to incorporate straw into their fields are key factors influencing adoption decisions (Sattler *et al*., 2010; Lu *et al*., 2020).

A pilot study conducted by the ICAR-Indian Institute of Soil Science to examine the diffusion and adoption of recommended nutrient management practices in the soybean-wheat rotations revealed wide variability in the on-farm resource base of smallholder farmers. It also underscored the critical role of farmer participation in the development and evaluation of agricultural technologies. Recognizing these complexities, a study was undertaken using a Participatory Technology Development (PTD) approach in the soybean–wheat rotation system of Madhya Pradesh for a period of three years. This research aimed to determine how nutrient management practices and the efficiency with which farmers utilize available on-farm resources significantly influence yield and income in the soybean–wheat rotation system under varying environmental conditions in India. ~~The study assumed that, under varying environmental conditions, yield and income in soybean–wheat rotation system in India is significantly influenced by nutrient management practices and the efficiency with which farmers utilize available on-farm resources~~.

Formatted: Font: Italic

Formatted: Font: Italic

Formatted: Font: Italic

Formatted: Font: Italic

Materials and Methods

Study area, experimental design and treatments

The study was conducted in the Berasia region, situated in Bhopal district, Madhya Pradesh, India. Geographically, the region is located at 23.6830253°N and 77.4773669°E, with an elevation of 470 meters at the eastern edge of the Malwa Plateau in central India. The climate is classified as subtropical, with most of the rainfall occurring during the southwest monsoon season. The mean annual temperature is 25°C, and annual rainfall ranges between 850 mm and 1800 mm. Predominant cropping systems in the area include soybean–wheat and soybean–chickpea rotations, with maize and rice cultivated as rainfed crops in certain pockets. Most farmers belong to marginal to medium landholding categories (Government of India, 2020). ~~Approximately 80% of the population in Madhya Pradesh engages in agriculture, with cultivators comprising this significant demographic. Agriculture utilizes around 58% of the total land area. Most farmers in the region belong to marginal to medium landholding categories, managing approximately 95% of agricultural land (Government of India, 2020). Predominant cropping systems include soybean-wheat and soybean-chickpea rotations, with maize and rice cultivated as rainfed crops in some areas.~~

An initial survey revealed low economic profitability attributed to suboptimal nutrient management practices. ~~The pilot survey that acted as a lead to the study revealed low economic profitability due to suboptimal land management practices in the study area~~. Farmers typically applied only nitrogenous and phosphatic fertilizers, with farmyard manure (FYM) applications limited to 1.5-2 t ha^{-1} every 2-3 years based on availability. Although high yielding varieitis such as ~~The study showed that though high-yielding~~ soybean ~~variety~~ JS-355 (yield potential ~1.89 t ha^{-1}) and wheat ~~varieties~~ Lok 1 and HI8498 (*Malav Shakti*) (yield potential >4.5 t ha^{-1}) were adopted by the farmers crop yields were only ~~averaged to~~ 1.2 t ha^{-1} for soybean and 2.5 t ha^{-1} for wheat, which clearly indicated inadequate nutrient management. ~~indicating inadequate nutrient management~~. To identify optimal nutrient management for maximizing yield and economic returns, ~~from the soybean-wheat system,~~ Participatory Technology Development

Formatted: Font: Not Bold

Formatted: Font: Italic

(PTD) approach ~~-~~was implemented across nine selected farms. Selection criteria for farmers included managerial capability and willingness to allocate resources and support trials. Farms were also selected in such a way that they equally represented landholding categories ~~-~~small ~~holdings~~ (1-2 ha), medium ~~holdings~~ (2-4 ha), and semi-medium ~~holdings~~ (4-10 ha) categories. For the field trials, four nutrient management practices were defined for the soybean-wheat crop rotation system as follows.

1. T_1 (INM_{FYM}): Integrated Nutrient Management (INM) with recommended 5 t ha^{-1} FYM, *Rhizobium* biofertilizers (R33 & R34, 4 kg ha^{-1}), and 50% NPK for soybean; PGPR biofertilizers (P3 & P10, 4 kg ha^{-1}) and 75% NPK for wheat.
2. T_2 (INM_{EC}): INM developed in a participatory mode integrating enriched compost (phospho-sulpho-nitro-compost) at 2 t ha^{-1}, substituting 5 FYM t ha^{-1} of the T_1 (INM_{FYM}), promoting on-farm residue recycling using wheat straw.
3. T_3 (STCR): Soil Test Crop Response ~~test based~~ inorganic input recommendations developed for the region through All India Coordinated Research Project on Soil Test Crop Response correlation (ICAR). ~~of Indian Council of Agricultural Research~~. ~~Here, soil testing was done for the nine farmlands and specific target yield based nutrient recommendations were diploid in each farm based on its initial soil test values~~. Nutrient recommendations were calculated for each of the nine farm plots based on their initial soil test values to achieve specific target yields.
4. T_4 (FP): Farmers' practice involving 1 t ha^{-1} FYM with nitrogenous and phosphatic fertilizers.

~~T_1 and T_2 treatments were formulated considering the 100% NPK recommendation for soybean and wheat crops in their rotation system in Madhya Pradesh i.e~~The Recommended Dose of Fertilizer (RDF) used as the baseline for the INM treatments (T_1 and T_2) was: ~~,~~ 20 kg ha^{-1} N, 60 kg ha^{-1} P_2O_5 and 20 kg ha^{-1} K_2O ~~to~~ for soybean crop and 120 kg ha^{-1} N, 60 kg ha^{-1} P_2O_5

Formatted: Font: Italic

Formatted: Font: Not Bold

Formatted: Font: Not Bold

Formatted: Subscript

Formatted: Subscript

and 40 kg ha^{-1} K_2O for wheat crop. Biofertilizers were applied in dry and liquid forms through seed treatments. The experiment followed a Randomized Block Design (RBD) in an on-farm setting, with each of the nine selected farmers serving as a replication. Treatment plots were large (1000 m² each), leading to a total evaluated area of 3.6 hectares. ~~The experimental design was randomized block design (RBD), with each farmer serving as a replication. Treatment plots were each 1000 m², totalling 3.6 hectares under evaluation.~~

Climatic parameters

~~Regular observations were recorded for key climatic factors influencing the growth and development of soybean and wheat crops during the *Kharif* (June–July to October–November) and *Rabi* (October–November to March–April) seasons over a span of three years~~. Climatic factors influencing crop growth were monitored over the three-year study during the *Kharif* (June–July to October–November) and *Rabi* (October–November to March–April) seasons. Parameters such as maximum temperature, minimum temperature, average daily temperature and rainfall amount and distribution were monitored, particularly during the critical growth stages of these crops. ~~These climatic data were then correlated~~ These data were then subjected to correlation analysis with crop yields to evaluate the influence of weather conditions on overall productivity.

Soil analysis

Surface soil samples (0-15 cm depth) were collected pre- trial (before the first crop season)- and post-trial (after three years) from the nine farm fields. Initial composite soil samples were derived from five points per 4000~~-~~m² area to established baseline conditions.~~plot~~. The ~~S~~soil samples taken pre-trial from each treatment plot~~ before the first crop season and~~ were analysed for the physical and chemical properties.

Formatted: Font: Italic

Formatted: Font: Italic

Formatted: Font: Italic

Formatted: Font: Italic

Initial ~~The~~ soil properties for the ~~of the~~ selected agroecosystem averaged as 45% sand, 13.5% silt and 41.5% clay content (Loam texture) with pH of~~–~~ 7.63, Electricity Conductivity (EC) of – 0.2 ds m^{-1}, organic carbon - 6.3 g kg^{-1}, available nitrogen - 204.9 kg ha^{-1}, available phosphorus - 12.3 kg ha^{-1}, available potassium - 269 kg ha^{-1}. Specific nutrient recommendations for ~~(~~T_3~~)~~ were based on these ~~for the nine fields were based on these~~ initial soil test values.

Intercultural operations

~~To carry out the farm field evaluations of the nutrient management technologies a cost sharing approach was adopted. Inputs provided included seeds, chemical and biofertilizers, compost preparation materials (portable beds, rock phosphate, iron pyrite), and plant protection chemicals~~ A cost-sharing approach was adopted to facilitate the field evaluation, with the study covering the cost of new inputs (seeds, chemical and biofertilizers, compost preparation materials (portable beds, rock phosphate, iron pyrite], and plant protection chemicals). Farmyard Manure (FYM) for T_1 and T_4 was sourced on-farm.

The soybean variety used was the early maturing JS 95-60 (average yield ~1.6 t ha^{-1}), and the wheat variety GW-322 (average yield ~4.5 t ha^{-1} even under late sowing). Seed rates were 80 kg ha^{-1} for soybean and 100 kg ha^{-1} for wheat for all treatments. Soybean sowing occurred mid-June to early July, contingent on monsoon onset, with wheat sowing 3-4 weeks post-soybean harvest in October. Wheat irrigation frequency varied based on water availability (3-5 irrigations). Soybean received basal NPK fertilizers; wheat received 50% basal nitrogen, full P and K, with remaining N top-dressed in two split doses along with irrigation. For the wheat crop, quantity and frequency of irrigations was based on water availability in the on-farm water resources, even though the crop received at least three irrigations in season. Herbicide (Imazethapyr 10% SL, 750 ml ha^{-1}) and pesticide (Triazophos 40 EC, 250 gm ha^{-1} AI) were applied to soybean crop as needed.~~.~~

Total system productivity and economic analysis

~~Crop yields (grain and straw) were measured when 80 percent or more of the crop plants got dried after attaining maturity~~Yields of grain and straw for both crops were recorded when crop plants attained physiological maturity. For yield estimation, one random ~~sampling of~~ 4 m² quadrat sample ~~area within each treatment plot across nine farms~~ was collected from each treatment plot across the nine farms, and the resulting yields ~~for both soybean and wheat crops. The yields obtained~~ were ~~then~~ extrapolated to kg ha^{-1}. The total system productivity of the soybean–wheat cropping system was evaluated by calculating the system yield. This involved converting the soybean yield into ~~w~~Wheat E~~e~~quivalent Y~~y~~ield (WEY) using the respective annual market prices of the two crops. The wheat equivalent yield of soybean was then summed with the actual wheat yield to derive the overall system yield. An economic analysis was conducted to determine the gross returns and total production costs, deriving a benefit-cost ratio. Production costs accounted for machinery use, seeds, manures and fertilizers, herbicides, and plant protection chemicals. Input prices and Minimum Support Prices (MSP) for soybean and wheat were tracked annually to assess farm-level economics. To evaluate the yield stability and consistency of the soybean–wheat system under different nutrient management practices, the Sustainable Yield Index (SYI) as proposed by Singh *et al*. (1990) was used.

Formatted: Font: Italic

$$SYI = (\bar{Y} - \sigma) / Y_{max}$$

Where: $\bar{\mathbf{Y}}$ = Mean yield over the years, $\boldsymbol{\sigma}$ = Standard deviation (a measure of risk or yield variability) and $\mathbf{Y_{max}}$ = Maximum observed yield. Here, low values of standard deviation and higher value of SYI indicates that the system is more sustainable.

Nutrient balance calculation

Nutrient balances were computed for major nutrients (NPK), considering inputs and outputs in kg ha^{-1} year^{-1}. Negative balances indicated nutrient depletion, while positive balances indicated

accumulation. The average annual nutrient addition to the soybean-wheat system through manures and fertilizers under different nutrient management practices are given in Table 1.

Table 1. Average annual nutrient addition to soybean-wheat system under the four treatments

Treatments	Average nutrient addition in each crop season (kgha^{-1})						
	Organic manure				Inorganic fertilizers		
	N	P_2O_5	K_2O	S	N	P_2O_5	K_2O
**T_1** (INM_{FYM})	25.0	10.0	25.0	0.0	100.0	75.0	40.0
**T_2** (INM_{EC})	20.0	40.0	42.0	34.0	100.0	75.0	40.0
**T_3** (STCR)	0.0	0.0	0.0	0.0	156.3	188.0	91.6
**T_4** (FP)	5.0	2.0	5.0	0.0	100.0	145.0	0.0

Statistical analysis

Data collected over three crop seasons were subjected to Combined Analysis of Variance statistical analysis using analysis of variance (ANOVA) appropriate to the experimental design for various agronomic and economic parameters. A pooled (combined) analysis was conducted for yield and economic indicators to evaluate treatment effects across years. For comparing treatment means, post hoc analysis was carried out using Tukey's Honest Significant Difference (HSD) test at a significance level of P = 0.05 to determine statistically distinct groups (t-grouping) for each parameter. All statistical analyses were performed using SAS software (Version 9.3), employing the Generalized Linear Model procedure (PROC GLM). Treatment means were compared using Tukey's Honest Significant Difference (HSD) test at a significance level of $P < 0.05$. All statistical analyses were performed using SAS software (Version 9.3), employing the Generalized Linear Model procedure (PROC GLM).

Results and Discussion

Climatic parameters

~~Climatic parameters of the study area during the three soybean -wheat rotation periods (Figure 1) indicate a distinct monsoon pattern, with peak rainfall occurring consistently in July each year~~ Climatic data recorded over the three years (Figure 1) indicated a distinct unimodal rainfall pattern characteristic of the region, with peak precipitation consistently occurring in July, ranging between 500 to 600 mm. This high-~~-~~rainfall period (~~, extending from~~ June to September), coincided with elevated maximum temperatures (30 to ~~–~~36°C), ~~creating optimal conditions for soybean cultivation~~ during the *Kharif* season. Since the soybean variety used for the study was an early maturing one, duration of the crop was less than 100 days. ~~and~~ S~~s~~owing was ~~done~~ carried out after the effective onset of the monsoon (6–7 initial rainy days).

Fig 1. Climate Graph of all the three soybean-wheat crop rotation seasons during the study period

Temperature in the first *Kharif* season showed average seasonal temperature (T_{avg}) of 27°C with maximum (T_{max}) and minimum (T_{min}) ~~-~~monthly average ~~(T_{max})~~ of 33.6°C in June and ~~minimum monthly average (T_{min}) of~~ 22.1°C in September, respectively. The ~~M~~monsoon start ~~hit~~ in the region by the second week of June recorded 1473.0 mm total seasonal rainfall ~~and that was well~~ distributed over 76 rainy days. However, the second *Kharif* season observed a slight change in the weather pattern due to the late monsoon arrival by the second week of July. The season recorded ~~showed~~ a T_{avg} value of 28.9°C, T_{max} of 33.1°C in the month of July and T_{min} of 19.2°C in the month of October. Moreover, the season received a weak rainfall of 858.7 mm ~~that was~~ distributed over 59 rainy days. Although the third *Kharif* season followed a similar temperature pattern as that of the first season with a T_{avg},T_{max}, T_{min} values of 27.7°C, 33.9°C,

Formatted: Subscript

Formatted: Subscript

and 21.1°C respectively., The monsoon was not evenly distributed with a monsoon showers were not well distributed. The total seasonal rainfall of 1192.9 mm was spread over a short period of 51 rainy days.

The graph shows that from October onward, both rainfall and temperatures began to decline in all the three seasons, with the *Rabi* season (October to April) characterized by minimal rainfall and cooler temperatures, especially in December and January when minimum temperatures dropped to 4– to 6°C. During the first *Rabi* season the average (T_{avg}) maximum (,T_{max}) and minimum (,T_{min}) values recorded for the first *Rabi* season were respectively 22.3°C, 29.2°C (October), and 10.3°C (January), respectively. During the second season the same were recorded as and that of second season were 22.1°C, 29.5°C (November), and 8.3°C (January) 24(January) 24.2°C, 31.9°C (November), and 10.6°C (December). These conditions are conducive to wheat growth, which typically benefits from dry weather and moderate temperatures during its vegetative and reproductive stages.

The southwest monsoon, which typically arrives in the Bhopal district by mid-June and continues until the end of September, plays a crucial role in ensuring water availability during the critical growth stages of the soybean crop, thereby supporting improvinged the seed yield. Although the third crop season experienced substantial rainfall from June to August, a prolonged dry spell of 12 days in September coincided with the pod-filling stage of soybean. Drought stress during the pod development and filling stages can significantly reduce yield by decreasing the number of seeds per pod and resulting in smaller seed size (De Souza *et al.*, 1997; Gebre and Earl, 2020; Zeleke and Nendel, 2024). Since groundwater availability for irrigation is influenced by seasonal precipitation patterns (Asoka and Mishra, 2020), variability in the arrival and withdrawal of the monsoon—as well as the intensity and distribution of rainfall during the study period—may have adversely affected the growth and yield of rainfed

Formatted: Font: Italic

Formatted: Not Superscript/ Subscript

Formatted: Not Superscript/ Subscript

Formatted: Not Superscript/ Subscript

Formatted: Font: Italic

soybean, and consequently the wheat crop, particularly on farms with limited on-farm water resources.

Overall,~~ the climatic pattern during the study period supported the successful implementation of the soybean–wheat system, with climatic variability playing a notable role in influencing productivity~~ the variability in the arrival and withdrawal of the monsoon, along with the intensity and distribution of rainfall during the study period, likely played a notable role in influencing the productivity of the rainfed soybean crop and, consequently, the succeeding wheat crop, particularly on farms with limited on-farm water resources. The overall climatic pattern during the study period supported the successful implementation of the soybean–wheat system.~~.~~

Impact of different nutrient management practices on crop yield

The yield performance of soybean and wheat under different treatments during the three crop rotation seasons revealed significant effects of year, treatment, and their interaction on crop productivity and sustainability (Table 2).

Table 2. Average yield and Sustainable Yield Index (SYI) of soybean and wheat ~~crops~~ under different nutrient management practices in the soybean–wheat crop rotation system.

Crop	Treatments	Average Yield (t ha^{-1})				SYI
		**Year 1**	**Year 2**	**Year 3**	**Pooled**	
**SOYBEAN**	**T$_1$** (INM$_{FYM}$)	1.14a	1.15a	0.91c	1.07b	0.82
	**T$_2$** (INM$_{EC}$)	1.25a	1.22a	1.05a	1.17a	0.86
	**T$_3$** (STCR)	1.19a	1.17a	1.00b	1.12ab	0.85

	**T_4** (FP)	1.04a	0.98b	0.83d	0.95c	0.81
Sources of variation		*Probability level of significance*				
Year		-	-	-	*<0.0001*	
Treatment		*0.1345*	*0.0004*	*<0.0001*	*<0.0001*	
Year x Treatment		-	-	-	*<0.0001*	
**WHEAT**	**T_1** (INM$_{FYM}$)	4.06a	3.79b	3.90c	3.92b	0.93
	**T_2** (INM$_{EC}$)	4.39a	4.20a	4.25a	4.28a	0.95
	**T_3** (STCR)	4.13a	3.96b	4.08b	4.06b	0.96
	**T_4** (FP)	3.56b	3.42c	3.67d	3.55c	0.93
Sources of variation		*Probability level of significance*				
Year		-	-	-	*<0.0001*	
Treatment		*0.0003*	*<0.0001*	*<0.0001*	*<0.0001*	
Year x Treatment		-	-	-	*<0.0001*	

Means followed by the same letter/letters are not significantly different based on Tukey's Honest Significant Difference (HSD) at probability P=0.05.

For soybean, treatment T_2 (INM-EC) consistently recorded the highest yields across the three years, with a pooled average of 1.17 t ha^{-1} and the highest Sustainable Yield Index (SYI) of 0.86. T_3 (STCR) followed closely with a pooled yield of 1.12 t ha^{-1} and SYI of 0.85. Treatment T_4 (Farmers' Practice) recorded the ~~consistently showed the~~ lowest performance, with a pooled yield of 0.95 t ha^{-1} and the lowest SYI (0.81). Although treatment differences were not significant in the first year, they became statistically significant in subsequent years and in the pooled data, indicating a growing treatment impact over time. The significant Year × Treatment interaction ($p < 0.0001$) suggests that treatment effects varied considerably with climatic

conditions, especially during the third year which recorded comparatively lower yields. ~~likely influenced by climatic variability observed in that year~~.

In wheat, treatment T_2 again emerged as the top performer with a pooled average yield of 4.28 t ha^{-1} and a high SYI of 0.95. T_3 also maintained strong ~~consistent~~ performance with a pooled yield of 4.06 t ha^{-1} and the highest SYI among all treatments at 0.96, indicating strong yield stability across years. T_4 had the lowest yield (3.55 t ha^{-1}), although it maintained a relatively high SYI of 0.93, indicating that while its productivity was lower, it was more consistent. Unlike soybean, treatment differences in wheat were significant in all individual years and the pooled analysis ($p < 0.0001$), highlighting a stronger response of wheat to treatment interventions. Again, the significant Year × Treatment interaction confirms that seasonal climatic conditions influenced treatment effectiveness.

Overall, ~~T_2 that integrated enriched compost and biofertilizers with chemical fertilizers proved to be the most productive and sustainable treatment for both crops, with high yields and stable performance over three years. The results underscore the importance of treatment choice in maintaining yield stability, especially under variable climatic conditions, as reflected in the SYI values~~ the superior performance of the nutrient management treatments T_2 (INM_{EC}) and T_3 (STCR) was consistent across both soybean and wheat in terms of yield and SYI. T_2 provided the highest overall productivity in both crops, while T_3 demonstrated the highest stability in wheat (SYI of 0.96). These findings underscore the importance of optimal nutrient management in maintaining yield stability under variable climatic conditions compared to the Farmers' Practice, as reflected in the SYI ~~.~~These findings support the recommendation of T_2 as a preferred management strategy in the soybean–wheat system for achieving both productivity and resilience. Yield advantages resulting from the use of enriched compost (phospho-sulpho-nitro compost) for soybean and wheat crops have also been reported by Manna *et al*. (2001) and Pal *et al*. (2020).

Formatted: Font: Italic

Formatted: Font: Italic

Variation in crop yield across the farm holdings

The average seed yield of soybean and wheat crops from different nutrient management practices (Figure 2 and 3) showed notable variability across the farm holdings and years. As shown in Figure 2, ~~It is evident from the graph (Figure 2) that the~~ treatment T_2 consistently produced the highest average soybean seed yields across most farm sizes in all three years, recording the ~~and recorded~~ highest yield of 1.6 t ha^{-1} in the first crop season. Soybean yields were generally higher in larger holdings (semi-medium), followed by medium and small holdings, suggesting a possible advantage of resource availability or management efficiency in larger farms. Treatment T_4 consistently recorded the lowest yields across all holding categories and years, highlighting its limited effectiveness. The first year showed the most favourable yield performance overall, followed by second year, with a noticeable decline in the third year, likely influenced by climatic variations such as reduced rainfall or temperature extremes, as seen in the earlier climate graph.

For the wheat crop (Figure 3) the average grain yield across different farm holding sizes and treatments ~~during the study period~~ demonstrated consistent trends, with Treatment T_2 producing the highest yields across most categories. Semi-medium holdings consistently achieved the highest yields, peaking near or above 4.8 t ha^{-1}, indicating more efficient resource utilization and possibly better access to inputs or mechanization. Across all years, T_4 recorded the lowest yields, especially in small and medium holdings, reinforcing its relatively poor agronomic performance. Year-wise, the second crop season showed slightly superior overall grain yield compared to the other years, with T_2 maintaining a lead regardless of farm size. Notably, the difference in yields among holding types narrowed slightly in the third year, perhaps reflecting uniform environmental stress or improved management practices across holdings.

The error bars across categories in both graphs indicate a moderate variability, with greater consistency in both soybean and wheat crop yields from T_2, developed through a participatory technology development approach. These trends emphasize that T_2 is the most effective treatment for enhancing yield from the soybean-wheat rotation system irrespective of holding size likely due to the better adaptability to recommended practices. The semi-medium holdings consistently outperform smaller categories, possibly due to better input access and agronomic practices.

Fig 2. Yield of soybean ~~crop~~ across farm holdings from different nutrient management options during the study period

Fig 3. Yield of wheat ~~crop~~ across farm holdings from different nutrient management options during the study period

Economic performance of the soybean-wheat rotation system

An integrated assessment of yield, cost of cultivation, gross and net income, and benefit-cost ratio (BCR) across four treatments in the soybean–wheat system reveals significant difference between treatment on both crop productivity and economic returns over the three-year period (Table 3).

Table 3. Economic aspects of soybean, wheat and soybean-wheat system (average of 3 years)

Crop	Treatments	System Yield	Cultivation Cost	Gross Income	Net Income	BCR

		(Pooled)	(**INR ha^{-1})	$^{(}$INR ha^{-1})	(INR ha^{-1})	
**SOYBEAN**	**T$_1$** (INM$_{FYM}$)	2.14b*	18603.00b	35236.67b	16633.67b	1.9c
	**T$_2$** (INM$_{EC}$)	2.35a*	20203.00a	38756.67a	18553.67b	1.9c
	**T$_3$** (STCR)	2.25ab*	14650.11c	37082.22ab	22432.11a	2.6a
	**T$_4$** (FP)	1.88c*	14750.00c	31313.33c	16563.33b	2.1b
Sources of variation			*Probability level of significance*			
Year		*<0.0001*	*<0.0001*	*<0.0001*	*<0.0001*	*<0.0001*
Treatment		*<0.0001*	*<0.0001*	*<0.0001*	*<0.0001*	*<0.0001*
Year x Treatment		*<0.0001*	*<0.0001*	*<0.0001*	*<0.0001*	*<0.0001*
**WHEAT**	**T$_1$** (INM$_{FYM}$)	3.92b	19300.00c	62666.67b	43366.67b	3.3b
	**T$_2$** (INM$_{EC}$)	4.28a	19300.00c	68426.67a	49126.67a	3.5a
	**T$_3$** (STCR)	4.06b	25347.67a	64911.41b	39563.74c	2.6c
	**T$_4$** (FP)	3.55c	21750.00b	56770.37c	35020.37d	2.6c
Sources of variation			*Probability level of significance*			
Year		*<0.0001*	*0.0035*	*0.0022*	*0.0056*	*0.0122*
Treatment		*<0.0001*	*<0.0001*	*<0.0001*	*<0.0001*	*<0.0001*
Year x Treatment		*<0.0001*	*<0.0001*	*<0.0001*	*<0.0001*	*<0.0001*
**SOYBEAN-**	**T$_1$** (INM$_{FYM}$)	6.05b	37903.00b	97903.33c	60000.33b	2.6ab
**WHEAT**	**T$_2$** (INM$_{EC}$)	6.63a	39503.00a	107183.33a	67680.33a	2.7a
**SYSTEM**	**T$_3$** (STCR)	6.30ab	39997.78a	101993.63b	61995.85b	2.6b
	**T$_4$** (FP)	5.43c	36500.00c	88083.70d	51583.70c	2.4c
Sources of variation			*Probability level of significance*			
Year		*<0.0001*	*<0.0001*	*<0.0001*	*<0.0001*	*<0.0001*

Treatment	*<0.0001*	*<0.0001*	*<0.0001*	*<0.0001*	*<0.0001*
Year x Treatment	*<0.0001*	*<0.0001*	*<0.0001*	*<0.0001*	*<0.0001*

Means followed by the same letter/letters are not significantly different based on Tukey's Honest Significant Difference (HSD) at probability P=0.05.

*** Wheat equivalent yield** **** 1 INR = 0.012 USD = 0.010 EUR**

In the case of soybean, the pooled average seed yield of three ~~consecutive~~ years was ~~recorded the~~ highest for T_2 ~~soybean yield~~ (1.17 t ha^{-1}), followed ~~closely~~ by T_3 (1.12 t ha^{-1}), with ~~hile~~ T4 recording ~~ed~~ the lowest (0.95 t ha^{-1}). Although T_2 recorded ~~had~~ the highest gross income (38,756.67 INR ha^{-1}), its net income (18,553.67 INR ha^{-1}) was lower than T_3 due to additional cost of off-farm inputs for the preparation of enriched compost. T_3~~,~~ emerged as the most economically efficient nutrient management practice for soybean, with the highest net income (22,432.11 INR ha^{-1}) and a superior Benefit Cost Ratio (BCR) of 2.6, owing to its relatively low cost of cultivation because of soil test-based fertilizer application. T_4~~,~~ despite having a low yield, maintained a moderate BCR of 2.1 due to its low cultivation cost.

For the ~~In the case of~~ wheat crop, T_2 again demonstrated the highest yield (4.28 t ha^{-1}) and generated the maximum net income (49,126.67 INR ha^{-1}) with the highest ~~best~~ BCR (3.5), highlighting its economic superiority. T_3, while yielding moderately (4.06 t ha^{-1}), was associated with a ~~suffered from a~~ high cultivation cost (25,347.67 INR ha^{-1}) due to the more fertilizer requirement, which resulted in a reduced ~~its~~ net income to 39,563.74 INR ha^{-1} and BCR to 2.6. T_4 had the lowest yield (3.55 t ha^{-1}) and net income (35,020.37 INR ha^{-1}), and its BCR also remained at 2.6. This indicates that while T_3 was cost-intensive, its returns did not proportionally increase.

~~While~~ ~~c~~Considering the ~~performance of~~ soybean–wheat rotation system, T_2 was the best performer with 6.63 t ha^{-1} in terms of the overall system yield, followed by T_3 (6.30 t ha^{-1}), with ~~and~~ T_4 recording the ~~had the~~ lowest at 5.43 t ha^{-1}. The highest gross (107,183.33 INR ha^{-1}) and net income (67,680.33 INR ha^{-1}) were also associated with T_2. T_3 maintained competitive performance in both productivity and profitability, while T_1 showed moderate results. Although T_4 had the lowest productivity and income, it achieved a reasonable BCR of 2.4, indicating some economic viability due to lower inputs. Furthermore, the statistical analysis confirmed that year, treatment, and year × treatment interactions significantly influenced all economic and yield parameters ($p < 0.0001$). This suggests that the year-to-year variations in rainfall intensity and distribution, particularly during the monsoon months, likely contributed to the significant differences observed in system yields and economic returns across treatments.

Impact of nutrient management practices on soil health

Nutrient budgeting of different nutrient management systems

The nutrient balance analysis for nitrogen (N), phosphorus (P), and potassium (K) under the soybean–wheat cropping system (Table 4) revealed ~~highlights~~ significant differences across the four ~~nutrient management~~ treatments. Treatment T_3 recorded ~~exhibited~~ the most balanced nutrient dynamics, with relatively lower negative balances for nitrogen (–19.2 kg ha^{-1}) and potassium (–19.3 kg ha^{-1}), along with the highest positive phosphorus balance (+52.9 kg ha^{-1}), indicating efficient nutrient utilization and minimal losses. In stark contrast, Treatment T_4 showed the most severe nutrient depletion, particularly for potassium (–104.3 kg ha^{-1}) and nitrogen (–52.5 kg ha^{-1}), likely due to inadequate potassium application (only 4 kg ha^{-1}). However, ~~Although~~ T_4 exhibited a higher phosphorus uptake than input, it still maintained a small positive P balance.

Treatment T_2, despite receiving higher nutrient inputs, recorded substantial deficits in N (–42.1 kg ha^{-1}) and K (–42.7 kg ha^{-1}), suggesting either poor nutrient uptake efficiency or higher crop demand, both of which may lead to long-term soil fertility degradation if not addressed. Treatment T_1 showed moderate performance, with ~~manageable~~ deficits in N (–17.7 kg ha^{-1}) and K (–57.0 kg ha^{-1}), and a modest P surplus (+19.4 kg ha^{-1}).

Table 4. Nutrient balance sheet of soybean-wheat crop rotation system under different nutrient management practices

Treatments	Nutrient applied to soil (kg ha^{-1})			Nutrient removal by crops (kg ha^{-1})			Apparent Nutrient Balance (kg ha^{-1})		
	**N**	**P**	**K**	**N**	**P**	**K**	**N**	**P**	**K**
**T_1** (INM_{FYM})	145	46	59	162.7	26.4	115.9	-17.7	19.4	-57.0
**T_2** (INM_{EC})	140	59	80	182.1	28.2	122.7	-42.1	30.7	-42.7
**T_3** (STCR)	148	80	86	167.2	27.1	105.3	-19.2	52.9	-19.3
**T_4** (FP)	97	51	4	149.5	31.7	108.3	-52.5	19.3	-104.3

These findings reinforce that balanced nutrient application, as demonstrated in T_3, promotes nutrient retention and system sustainability, while imbalanced or insufficient fertilization, as in T_4, accelerates nutrient mining and threatens soil health. Similar concerns over nutrient mining in Indian soils have been reported by Surendran *et al*. (2016), who advocate for integrated nutrient management strategies. Furthermore, Jayanthi~~y~~ *et al*. (2024) emphasized improved nutrient uptake efficiency under soil test–based nutrient application, supporting the observed benefits in T_3.

Formatted: Font: Italic

Formatted: Font: Italic

Effect of nutrient management practices on soil microbial populations

The average data on soil microbial populations (Table 5) reveal significant variations in the abundance of bacteria, fungi, and actinobacteria across the different nutrient management treatments. Bacterial populations (expressed as $\times 10^7$ cfu g^{-1} dry soil) were highest under Treatment T_3 (2.39), which was significantly greater than T_2 and statistically comparable to T_1, indicating enhanced bacterial activity potentially due to more balanced nutrient availability in T_3.

In contrast, fungal populations ($\times 10^4$ cfu g^{-1} dry soil) peaked in Treatment T_2 (3.46), showing a statistically significant increase over all the other treatments. This suggests that the nutrient regime in T_2 was particularly favorable for fungal proliferation, possibly due to specific organic inputs or microenvironmental conditions.

Table 5. Effect of different treatments on Soil microbial populations

**Treatments**	**Bacteria** **X10^7 cfu g^{-1} dry soil**	**Fungi** **X10^4 cfu g^{-1} dry soil**	**Actinobacteria** **X10^5 cfu g^{-1} dry soil**
**T_1** (INM_{FYM})	1.91ab	2.63b	3.01b
**T_2** (INM_{EC})	1.34b	3.46a	3.50a
**T_3** (STCR)	2.39a	2.09b	2.84b
**T_4** (FP)	1.45ab	1.30c	2.39c
Sources of variation	*Probability level of significance*		
Treatment	*0.0326*	*<0.0001*	*<0.0001*

Means followed by the same letter/letters are not significantly different based on Tukey's Honest Significant Difference (HSD) at probability P=0.05.

Actinobacterial counts (×10^5 cfu g^{-1} dry soil), which play a key role in organic matter decomposition and disease suppression, were also highest in T_2 (3.50), followed by T_1 and T_3. Treatment T_4 consistently recorded the lowest microbial counts across all groups—fungi (1.30), bacteria, and actinobacteria (2.39)—indicating a relatively less biologically active soil environment, possibly due to imbalanced nutrient inputs or suboptimal soil conditions.

The statistically significant treatment effects ($p < 0.05$) observed across microbial groups underscore the influence of nutrient management on the soil microbial ecology. Notably, T_2 enhanced both fungal and actinobacterial populations, while T_3 was more effective in promoting bacterial abundance. These findings highlight the potential of tailored nutrient strategies to improve soil biological activity and overall soil health in the soybean–wheat cropping system.

The importance of nutrient inputs—both organic and inorganic—in modulating soil microbial diversity and function has also been emphasized in earlier studies by Geisseler and Scow (2014), Zhou *et al*. (2015), and Yadav *et al*. (2018).

Formatted: Font: Italic

Formatted: Font: Italic

Conclusion

This study examined the impact of different nutrient management practices on productivity, profitability, and soil health under the soybean–wheat rotation system of Central India, amid increasing climatic variability. The findings clearly demonstrate that nutrient management plays a pivotal role in sustaining yield and income, particularly under changing climate conditions that challenge rainfed agriculture. Among the tested treatments, T_2—which combined enriched compost, biofertilizers, and chemical fertilizers—consistently delivered the

highest system yield (6.63 t ha^{-1}), net income (67,680.33 INR ha^{-1}), and benefit-cost ratio (3.5) while maintaining a high sustainable yield index. This treatment also significantly enhanced fungal and actinobacterial populations, indicating improved soil biological activity. T_3, with soil test–based nutrient application, emerged as the most nutrient-balanced system, promoting bacterial abundance and offering competitive economic and yield performance. Climatic variations, particularly in rainfall distribution and monsoon timing, strongly influenced crop performance, emphasizing the need for resilient nutrient management strategies. T_2 and T_3 proved more adaptable across diverse climatic scenarios and farm sizes, with semi-medium holdings achieving the best outcomes due to better input access.

Overall, the current research study emphasized this research underscores the on importance of integrated and balanced nutrient management in enhancing crop productivity, economic viability, and long-term soil health in the soybean–wheat system. The participatory and site-specific approaches adopted in T_2 and T_3 present scalable options for resource-limited farmers to adapt to climate change while ensuring sustainable agricultural intensification. Future research should focus on long-term nutrient cycling, water-use efficiency, and expanding these strategies to other agro-ecological regions to enhance system resilience.

Acknowledgments

The authors sincerely acknowledge the support of Indian Council of Agricultural Research to carry out this research. The cooperation of farmers of the study area is also gratefully appreciated.

References

~~1. Agarwal D K, Billore S D, Sharma A N, Dupare B U, Srivastava S K. Soybean: introduction, improvement, and utilization in India – problems and prospects. Agric. Res. 2013; 2(4), 293-300. https://doi.org/10.1007/s40003-013-0088-0.~~

~~2.~~1. Asoka A, Mishra V. A strong linkage between seasonal crop growth and groundwater storage variability in India. J. Hydrometeorol. 2020; 22,125–138. https://doi.org/10.1175/JHM-D-20-0085.1.

~~3. Asseng S, Ewert F, Martre P, Rotter R P, et al. Rising temperatures reduce global wheat production. Nat. Clim. Chang. 2015; 5, 143-147. https://doi.org/10.1038/nclimate2470.~~

~~4.~~2. Behera U K, Sharma A R, Pandey H N. Sustaining productivity of wheat-soybean cropping system through integrated nutrient management practices on the vertisols of central India. Plant and Soil 2007; 297:185-199. doi.org/10.1007/s11104-007-9332-3.

~~5.~~3. Caviglia O P, Sadras V O, Andrade F H. Yield and quality of wheat and soybean in sole and double cropping. Agron. J. 2011; 103(4), 1081-1089. doi.org/10.2134/agronj2011.0019.

~~6.~~4. De Souza P I, Egli D B, Bruening W P. Water stress during seed filling and leaf senescence in soybean. Agron. J. 1997; 89, 807-812. https://doi.org/10.2134/agronj1997.00021962008900050015x.

~~7.~~5. Dubey M, Shinogi K C, Awasthi S K, Khan MA. Comparing the nutrient management pattern in soybean and rice based cropping systems by soil health card holders and non-holders. Indian J. Ext. Educ. 2022; 58(3), 83-87. https://doi.org/10.48165/IJEE.2022.58318.

~~8.~~6. Dupare B U, Sharama P, Verma S K, Billore S D. Adoption of soybean production technology by the farmers in Malwa plateau of Madhya Pradesh. Soybean Research 2019; 17(1&2), 62-76.

~~9.~~7. Gebre M H, Earl H J. Effects of Growth Medium and Water Stress on Soybean [*Glycine max* (L.) Merr.] Growth, Soil Water Extraction and Rooting Profiles by Depth in 1-m Rooting Columns. Front. Plant. Sci. 2020; 11, 487. https://doi.org/10.3389/fpls.2020.00487.

~~10.~~8. Geisseler D, Scow K M. Long-term effects of mineral fertilizers on soil microorganisms–A review. Soil. Biol. Biochem. 2014; 75, 54–63. https://doi.org/10.1016/j.soilbio.2014.03.023

~~11.~~9. Government of India. All India Report on Agriculture Census 2015-16., Department of Agriculture & Farmers Welfare. 2020. https://agcensus1.da.gov.in/tehsilsummarytype.aspx.

~~12.~~10. Government of India. Agricultural Statistics at a Glance 2022. Ministry of Agriculture & Farmers welfare. 2023. https://agriwelfare.gov.in/Documents/CWWGDATA/Agricultural_Statistics_at_a_Glance_2022_0.pdf.

~~13. Gupta A, Singh C, Kumar V, Tyagi B S, Tiwari V, Chatrath R, et. Al. Wheat Varieties Notified in India since 1965. ICAR- Indian Institute of Wheat and Barley Research, Karnal, India. 2018. https://www.aicrpwheatbarleyicar.in/wp-content/uploads/2021/06/wheat-varieties-notified-in-india.pdf.~~

~~14.~~11. ICAR-IISR. Director's Report of AICRP on Soybean 2020-21. ICAR-Indian Institute of Soybean Research, Indore, Madhya Pradesh, India. 2021. https://iisrindore.icar.gov.in/pdfdoc/AicrpsAR2020-21.pdf.

~~15.~~12. Jain N, Bhatia A, Pathak H. Emission of air pollutants from crop residue burning in India. Aerosol Air Qual. Res. 2014; 14, 422-430. https://doi.org/10.4209/aaqr.2013.01.0031.

~~16.~~13. Jayanthi T, Latha H S, Madappa S, Umilsingh N, Umesh H R, Ashok LB. Budgeting of primary nutrients in soil under rice-cowpea cropping sequences in Alfisols. J. Adv. Biol. Biotechnol. 2024; 27 (10), 1046-61. https://doi.org/10.9734/jabb/2024/v27i101527

~~17.~~14. Kumar P, Kumar S, Joshi L. Socioeconomic and Environmental Implications of Agricultural Residue Burning. Springer Briefs in Environmental Science. Springer, New Delhi. 2015. https://link.springer.com/chapter/10.1007/978-81-322-2014-5_2.

~~18.~~15. Kundu S, Bhattacharyya R, Prakash V, Gupta H S, Pathak H. Ladha J K. Long term yield trend and sustainability of rainfed soybean-wheat system through farmyard manure application in a sandy loam soil of the Indian Himalayas. Biol. Fertil. Soils. 2007; 43, 271-280. https://link.springer.com/article/10.1007/s00374-006-0102-9.

~~19.~~16. Kushwah S S, Reddy D D, Somasundaram J, Srivastava S, Khamparia R S. Crop residue retention and nutrient management practices on stratification of phosphorus and soil organic carbon in the soybean–wheat system in vertisols of Central India. Commun. Soil. Sci. Plant. Anal. 2016; 47, 2387-2395. https://www.tandfonline.com/doi/full/10.1080/00103624.2016.1243703.

~~20.~~17. Leff B, Ramankutty N, Foley J A. Geographic distribution of major crops across the world, Global Biogeochem. Cycles. 2004; 18, 1-27. doi.org/10.1029/2003GB002108.

~~21.~~18. Lu H, Hu L, Zheng W, Yao S, Qian L. Impact of household land endowment and environmental cognition on the willingness to implement straw incorporation in

China. J. Clean. Prod. 2020; 262, 121479. https://doi.org/10.1016/j.jclepro.2020.121479.

~~22.~~19. Manna M C, Ghosh P K, Ghosh B N, Singh K N. Comparative effectiveness of phosphate-enriched compost and single superphosphate on yield, uptake of nutrients and soil quality under soybean - Wheat rotation. J. Agric. Sci. 2001; 137, 45-54. https://doi.org/10.1017/S0021859601008942.

~~23.~~20. Meena B P, Biswas A K, Singh M, Chaudhary R S, Singh A B, Das H, et.al. Long-term sustaining crop productivity and soil health in maize–chickpea system through integrated nutrient management practices in Vertisols of central India. Field. Crops. Res. 2019; 232, 62-76. https://www.sciencedirect.com/science/article/pii/S0378429018314096.

~~24.~~21. Morrison M J, Cober E R, Gregorich E, Voldeng H D, Ma B, Topp G C. Tillage and crop rotation effects on the yield of corn, soybean and wheat in eastern Canada. Can. J. Plant. Sci. 2018; 98(1), 183-191. doi/10.1139/CJPS-2016-0407.

~~25. Nagamani S, Tripathi A, Lal S K, Kumar A, Mandal B, Jain R K. First Report of Soybean yellow mottle mosaic virus on Soybean (Glycine max) in India. Plant. Dis. 2018; 102(8), 1673. doi.org/10.1094/PDIS-01-18-0080-PDN.~~

~~26.~~22. Pal A, Deshmukh P W, Chaure P R, Bhoyar S M. Assessing comparative effectiveness of phosphocompost and nitro-phospho-sulpho compost on yield, nutrient uptake of soybean crop and soil fertility on Vertisols. Journal of Pharmacognosy and Phytochemistry 2020; 9(3), 2272-2276. https://www.phytojournal.com/archives/2020/vol9issue3/PartAK/9-3-367-639.pdf.

~~27.~~23. Reddy D D, Kushwaha S, Srivastava S, Khamparia RS. Long-term wheat residue management and supplementary nutrient input effects on phosphorus

fractions and adsorption behavior in a vertisol. Commun. Soil. Sci. Plant. Anal. 2014; 45(4), 541-554. https://doi.org/10.1080/00103624.2013.867043.

~~28.~~24. Reddy K S, Mohanty M, Rao D L N, Subba Rao A, Pandey M, Singh M, et. al. Farmer involvement in the development and adoption of improved nutrient management technologies using the mother–baby trial approach in vertisols. Proc. Natl. Acad. Sci. India Sect. B Biol. Sci. 2015; 85, 51-62. https://doi.org/10.1007/s40011-013-0243-1.

~~29.~~25. Sattler C, Nagel U J. Factors affecting farmers' acceptance of conservation measures-A case study from north-eastern Germany. Land Use Policy. 2010; 27, 70-77. https://doi.org/10.1016/j.landusepol.2008.02.002.

~~30.~~26. Sharma P. Costs, returns and profitability of soybean cultivation in India: trends and prospects. Economic Affairs. 2016; 61(3), 413-425. DOI:10.5958/0976-4666.2016.00052.8

~~31.~~27. Singh R P, Das S K, Rao U M B, Reddy M N. Towards Sustainable Dryland Agricultural Practices. Bulletin 1990; 5–9. Central Research Institute for Dryland Agriculture, Hyderabad

~~32.~~28. Srivastava S, Rao A S, Biswas A K. Results of the participatory diagnosis of constraints and opportunities (PDCO) based trials from the state of Madhya Pradesh, in: Rao A S, Srivastava S (Eds.), Development of Farmers' Resource based Integrated Plant Nutrient Supply Systems: Experiments of a FAO-ICAR-IFFCO Collaborative Project and AICRP on Soil Test Crop Response Correlation, Indian Institute of Soil Science, Bhopal, Madhya Pradesh, India. 2001; 10-49.

~~33.~~29. Surendran U, Subramoniam S R, Raja P, Kumar V. Murugappan V. Budgeting of major nutrients and the mitigation options for nutrient mining in semi-arid tropical

agro-ecosystem of Tamil Nadu, India using NUTMON model. Environ. Monit. Assess. 2016; 188, 250. https://doi.org/10.1007/s10661-016-5202-x.

~~34.~~30. United States Department of Agriculture. World Agricultural Production, Foreign Agricultural Service Circular Series, WAP 6-24 June 2024. https://apps.fas.usda.gov/psdonline/circulars/production.pdf.

~~35.~~31. Watson C A, Reckling M, Preissel S, Bachinger J, Bergkvist G, Kuhlman T, et. al. Grain legume production and use in European agricultural systems. Adv. Agron. 2017;144, 235–303. doi.org/10.1016/bs.agron.2017.03.003.

~~36.~~32. Yadav A N, Verma P, Kumar S, Sangwan P, Mishra S, Panjiar N, Saxena AK. Plant microbiomes and sustainable agriculture: Diversity and beneficial interactions. Microbiol. Res. 2018; 206, 131–143. https://doi.org/10.1016/j.micres.2017.08.001.

~~37.~~33. Yigezu Y A, El-Shater T, Boughlala M, Bishaw Z, Niane A A, Maalouf F, et. al. Legume-based rotations have clear economic advantages over cereal monocropping in dry areas. Agron. Sustain. Dev. 2019; 39, 58. https://doi.org/10.1007/s13593-019-0602-2.

~~38. Zaveri E, Lobell D B. The role of irrigation in changing wheat yields and heat sensitivity in India. Nat. Commun. 2019; 10, 4144 https://doi.org/10.1038/s41467-019-12183-9.~~

~~39.~~34. Zeleke K, Nendel C. Yield response and water productivity of soybean (Glycine max L.) to deficit irrigation and sowing time in south-eastern Australia. Agric. Water. Mang. 2024; 296: 108815. https://doi.org/10.1016/j.agwat.2024.108815.

~~40.~~35. Zhou J, Guan D, Zhou B, Zhao B, Ma M, Qin J, et. al. Influence of 34-years of fertilization on bacterial communities in an intensively cultivated black soil in northeast China. Soil. Biol. Biochem. 2015; 90, 42–51. https://doi.org/10.1016/j.soilbio.2015.07.005.

~~41.~~36. Zuo Y, Zhang F. Iron and zinc biofortification strategies in dicot plants by intercropping with gramineous species: A review. Sustain. Agric. 2009; 29, 571–582. https://doi.org/10.1051/agro:2008055.

Responses to Reviewers

Dear Editor and Reviewers,

I am pleased to resubmit our revised manuscript entitled *"Soil fertility management for yield and income stability in soybean–wheat rotation system under climate variability in subtropical Central India"* (previously submitted as *"Soil fertility management for yield and income stability in soybean–wheat rotation system under changing climate in subtropical Central India"*), revised in accordance with reviewer suggestions, for further consideration in **PLOS ONE**.

I thank the Editor and Reviewers for their constructive feedback. The manuscript has been thoroughly revised to address all feedback. A detailed, point-by-point response document is provided below.

Reviewer #1:

Reviewer Edit Location	Action Taken	Justification
Page 1, Line 5	The title has been revised to "*Soil fertility management for yield and income stability in soybean-wheat rotation system under **climate variability** in subtropical Central India"*	The suggested term "varied climate" is broad and less technically precise. The revised wording "climate variability" better reflects the 3-year dataset, which captures inter-annual fluctuations in rainfall and temperature rather than long-term climate change trends.
Abstract	Suggested paraphrasing edits have been accepted.	Improved readability and scientific expression.
Introduction	Suggested paraphrasing and sentence restructuring edits have been accepted.	Enhanced clarity and coherence of the background and objectives.
Materials and Methods	Minor editorial edits suggested by the reviewer were accepted.	Improved consistency in terminology and readability.
Results and Discussion	Language edits incorporated as suggested.	Improved flow without affecting scientific interpretation.
References section	Suggested deletions were accepted; original reference formatting has been retained.	The manuscript already follows the journal's citation and reference formatting guidelines, and deleted references were not essential to support the study's objectives or discussion.

Reviewer #2:

Reviewer Edit Location	Action Taken	Justification
Line 144: Correct "diploid"	The term was removed during incorporation of Reviewer 1's corrections.	The word became redundant after revision and thus no longer appears in the updated manuscript.
Minor typographical errors noted	Typographical corrections have been made throughout the manuscript.	These edits improved accuracy and formatting consistency in the revised version.

Thank you again for these suggestions. We look forward to your positive response.

Yours Sincerely

Sanjay Srivastava
Principal Scientist
ICAR- Indian Institute of Soil Science
Nabibagh, Berasia Road, Bhopal, Madhya Pradesh, India- 462 038
Email:sanjaysrivastava238@gmail.com
Orcid: https://orcid.org/0009-0001-2935-4852
Google Scholar: https://scholar.google.com/citations?user=3ywrl7kAAAAJ&hl=en&authuser=2
