# Supplementary material for: Soil fertility management for yield and income stability in soybean-wheat rotation system under climate variability in subtropical Central India
Source: PLoS One. 2026 Jan 21;21(1):e0341035. doi: 10.1371/journal.pone.0341035 (PMC12822991; doi:10.1371/journal.pone.0341035)
Supplement: Attachment — Submitted filename: Response to Reviewers.docx [file pone.0341035.s005.docx]

**Responses to Reviewers**

**Dear Editor and Reviewers,**

I am pleased to resubmit our revised manuscript entitled *“Soil fertility management for yield and income stability in soybean–wheat rotation system under climate variability in subtropical Central India”* (previously submitted as *“Soil fertility management for yield and income stability in soybean–wheat rotation system under changing climate in subtropical Central India”*), revised in accordance with reviewer suggestions, for further consideration in **PLOS ONE**.

I thank the Editor and Reviewers for their constructive feedback. The manuscript has been thoroughly revised to address all feedback. A detailed, point-by-point response document is provided below.

**Reviewer #1:**

| **Reviewer Edit Location** | **Action Taken** | **Justification** |
| --- | --- | --- |
| Page 1, Line 5 | The title has been revised to “*Soil fertility management for yield and income stability in soybean-wheat rotation system under* ***climate variability*** *in subtropical Central India”* | The suggested term “varied climate” is broad and less technically precise. The revised wording “climate variability” better reflects the 3-year dataset, which captures inter-annual fluctuations in rainfall and temperature rather than long-term climate change trends. |
| Abstract | Suggested paraphrasing edits have been accepted. | Improved readability and scientific expression. |
| Introduction | Suggested paraphrasing and sentence restructuring edits have been accepted. | Enhanced clarity and coherence of the background and objectives. |
| Materials and Methods | Minor editorial edits suggested by the reviewer were accepted. | Improved consistency in terminology and readability. |
| Results and Discussion | Language edits incorporated as suggested. | Improved flow without affecting scientific interpretation. |
| References section | Suggested deletions were accepted; original reference formatting has been retained. | The manuscript already follows the journal’s citation and reference formatting guidelines, and deleted references were not essential to support the study’s objectives or discussion. |

**Reviewer #2:**

| **Reviewer Edit Location** | **Action Taken** | **Justification** |
| --- | --- | --- |
| Line 144: Correct “diploid” | The term was removed during incorporation of Reviewer 1’s corrections. | The word became redundant after revision and thus no longer appears in the updated manuscript. |
| Minor typographical errors noted | Typographical corrections have been made throughout the manuscript. | These edits improved accuracy and formatting consistency in the revised version. |

Thank you again for these suggestions. We look forward to your positive response.

Yours Sincerely

**Sanjay Srivastava**

Principal Scientist
ICAR- Indian Institute of Soil Science

Nabibagh, Berasia Road, Bhopal, Madhya Pradesh, India- 462 038

Email:[sanjaysrivastava238@gmail.com](mailto:sanjaysrivastava238@gmail.com)

Orcid: <https://orcid.org/0009-0001-2935-4852>

Google Scholar: <https://scholar.google.com/citations?user=3ywrl7kAAAAJ&hl=en&authuser=2>
